# RVD induction and autologous stem cell transplantation followed by lenalidomide maintenance in newly diagnosed multiple myeloma: a phase 2 study of the Finnish Myeloma Group

**DOI:** 10.1007/s00277-019-03815-7

**Published:** 2019-10-31

**Authors:** Sini Luoma, Pekka Anttila, Marjaana Säily, Tuija Lundan, Jouni Heiskanen, Timo Siitonen, Sakari Kakko, Mervi Putkonen, Hanna Ollikainen, Venla Terävä, Marja Sankelo, Anu Partanen, Kirsi Launonen, Anu Räsänen, Anu Sikiö, Merja Suominen, Piotr Bazia, Kristiina Kananen, Juha Lievonen, Tuomas Selander, Tarja-Terttu Pelliniemi, Sorella Ilveskero, Virva Huotari, Pentti Mäntymaa, Anri Tienhaara, Esa Jantunen, Raija Silvennoinen

**Affiliations:** 1 Comprehensive Cancer Center, Department of Hematology, Helsinki University Hospital and University of Helsinki, Helsinki, Finland; 2 Hematology-Oncology Unit, Oulu University Hospital, Oulu, Finland; 3 Department of Clinical Chemistry and TYKSLAB, University of Turku and Turku University Hospital, Turku, Finland; 4 Hematology Unit, Turku University Hospital, Turku, Finland; 5 Department of Medicine, Satakunta Central Hospital, Pori, Finland; 6 Hematology Unit, Tampere University Hospital, Tampere, Finland; 7 Department of Medicine, Kuopio University Hospital, Kuopio, Finland; 8 Department of Medicine, Mikkeli Central Hospital, Mikkeli, Finland; 9 Department of Medicine, Länsi-Pohja Central Hospital, Kemi, Finland; 10 Department of Medicine, Kymenlaakso Central Hospital, Kotka, Finland; 11 Department of Medicine, Central Finland Central Hospital, Jyväskylä, Finland; 12 Department of Medicine, Kanta-Häme Central Hospital, Hämeenlinna, Finland; 13 Department of Medicine, Kainuu Central Hospital, Kajaani, Finland; 14 Science Service Center, Kuopio University Hospital, Kuopio, Finland; 15 Fimlab Laboratories Ltd., Tampere, Finland; 16 HUSLAB Helsinki University Hospital, Helsinki, Finland; 17 NordLab Oulu, Oulu University Hospital, Oulu, Finland; 18 Laboratory of Eastern Finland, Kuopio University Hospital, Kuopio, Finland; 19 Institute of Clinical Medicine/Internal Medicine, University of Eastern Finland, Kuopio, Finland; 20 Department of Medicine, North Carelia Hospital District, Joensuu, Finland

**Keywords:** Multiple myeloma, Flow cytometry, PCR, Minimal residual disease, Lenalidomide, Maintenance

## Abstract

Autologous stem cell transplantation (ASCT) combined with novel agents is the standard treatment for transplant-eligible, newly diagnosed myeloma (NDMM) patients. Lenalidomide is approved for maintenance after ASCT until progression, although the optimal duration of maintenance is unknown. In this trial, 80 patients with NDMM received three cycles of lenalidomide, bortezomib, and dexamethasone followed by ASCT and lenalidomide maintenance until progression or toxicity. The primary endpoint was the proportion of flow-negative patients. Molecular response was assessed if patients were flow-negative or in stringent complete response (sCR). By intention to treat, the overall response rate was 89%. Neither median progression-free survival nor overall survival (OS) has been reached. The OS at 3 years was 83%. Flow-negativity was reached in 53% and PCR-negativity in 28% of the patients. With a median follow-up of 27 months, 29 (36%) patients are still on lenalidomide and 66% of them have sustained flow-negativity. Lenalidomide maintenance phase was reached in 8/16 high-risk patients but seven of them have progressed after a median of only 6 months. In low- or standard-risk patients, the outcome was promising, but high-risk patients need more effective treatment approach. Flow-negativity with the conventional flow was an independent predictor for longer PFS.

**Electronic supplementary material:**

The online version of this article (10.1007/s00277-019-03815-7) contains supplementary material, which is available to authorized users.

## Introduction

Autologous stem cell transplantation (ASCT) combined with novel agents is the standard treatment for transplant-eligible, newly diagnosed multiple myeloma (NDMM) patients. Bortezomib (V) plus dexamethasone (D) combined either with cyclophosphamide (CY), thalidomide, liposomal doxorubicin, or lenalidomide (R) are recommended for induction treatment [1]. RVD is not yet approved by the European Medicines Agency (EMA) for first-line treatment in transplant-eligible NDMM patients.

Lenalidomide maintenance after ASCT prolongs progression-free survival (PFS) [2–4] and overall survival (OS) [3] and is approved by EMA and Food and Drug Administration (FDA) for NDMM patients after ASCT until progression. The three randomized controlled trials (RCTs) evaluating lenalidomide maintenance were included in a meta-analysis [5] which demonstrated a significant PFS and OS benefit with lenalidomide maintenance after ASCT when compared with placebo or observation. The optimal duration of lenalidomide maintenance is a matter of debate. In Cancer and Leukemia Group B (CALGB) [3] and Intergroupe Francophone du Myelome (IFM) [2] trials, patients received post-ASCT placebo or continuous lenalidomide 10 mg for 3 months escalated then to 15 mg, until disease progression, intolerable side effects, or death. The IFM group decided to stop the treatment in the lenalidomide arm due to the noticed risk of secondary primary malignancies (SPMs) in opposite to the CALGB trial where patients continued maintenance until progression. Based on the meta-analysis, the risk of death due to SPM was similar between lenalidomide and placebo or observation groups but there was a 34% reduction in risk to die of multiple myeloma (MM) in lenalidomide maintenance arm [5].

Mian et al. showed in their retrospective analysis a positive correlation between the duration of lenalidomide maintenance and OS [6]. In the study by Goldschmidt et al., the patients were randomized to receive lenalidomide maintenance for 2 years or until CR. In patients receiving lenalidomide for 2 years, OS was higher [7].

The benefit of lenalidomide maintenance among high-risk (HR) patients has been demonstrated in Myeloma XI trial where the induction therapy was response-adjusted [8]. In Myeloma XI trial, 1q gain was also included in HR aberrations in addition to t(4;14), t(14;16), and del17p. Patients receiving lenalidomide during both induction and maintenance had the best outcome, and also, the HR patients gained benefit of lenalidomide maintenance. However, this was not as clear as in standard-risk (SR) patients and patients with 1q gain seemed to benefit more than the other HR patients.

The impact of minimal residual disease (MRD) negativity for PFS and OS has been demonstrated in several trials [9–14] and it is also recommended for one of the primary endpoints for evaluating the approval of new drugs for MM [9, 15].

This phase 2 trial of the Finnish Myeloma Group (FMG-MM02) was designed to investigate the rate of serological responses, proportion of flow-MRD-negative (10^-4^) patients and patients in molecular remission (10^-5^) after RVD induction followed by ASCT, and lenalidomide maintenance in NDMM patients. We also focused on the safety of RVD induction and lenalidomide maintenance and improvement of responses during the maintenance. The study included also a randomized stem cell mobilization with CY plus filgrastim or filgrastim alone in order to examine the success of stem cell mobilization after lenalidomide-based induction.

## Methods

### Patients

This study was nationally approved by the Research Ethics Committee of the Northern Savo Hospital District, and it was conducted according to the Declaration of Helsinki, International Conference of Harmonization and Guidelines for Good Clinical Practice. Written informed consent was obtained from all patients before inclusion. Key inclusion criteria were measurable, symptomatic, NDMM according to International Myeloma Working Group (IMWG) criteria [16, 17], age ≤ 70 years, and transplant-eligibility. Key exclusion criteria were peripheral neuropathy grade ≥ 2, significant liver dysfunction, severe cardiac dysfunction, severe renal failure (glomerular filtration rate < 15 ml/min, unless in hemodialysis), contraindication for the use of thromboprophylaxis or history of active malignancy during the past 5 years with the exception of basal cell carcinoma of the skin or stage 0 cervical carcinoma.

### Study design and treatment plan

This study was conducted at 12 centers in Finland. The study design (Online Resource 3) and protocol (Online Resources 4–5) are included in the supplementary material. The study is registered with number (NCT01790737) on clinicaltrials.gov and was monitored with level two by independent Clinical Research Units of University Hospitals of Finland.

Patients were initially treated with RVD induction comprising three 21-day cycles of lenalidomide 25 mg on days 1–14; bortezomib 1.3 mg/m^2^ on days 1, 4, 8, and 11 subcutaneously; and dexamethasone 20 mg/day on days 1–2, 4–5, 8–9, and 11–12. Acyclovir and enoxaparin prophylaxis were given during induction (Online Resources 4-5). The mobilization in arm A was CY 2 g/m^2^ on day + 1 plus filgrastim 5 μg/kg starting on day + 4, and in arm B, filgrastim 10 μg/kg alone starting on day + 1. The goal was to collect at least 3 × 10^6^/kg CD34+ cells for one transplant and 6 × 10^6^/kg if the second transplant was an option. The details of the apheresis procedure and guidelines for use of plerixafor as well as the results of the randomized mobilization study have been published previously [18]. Patients received a single ASCT after melphalan 200 mg/m^2^.

Lenalidomide maintenance was started 3 months after ASCT with a dose of 10 mg/day on days 1–21 in 28-day cycles and was continued until progression or toxicity. The threshold for start of lenalidomide and each cycle was neutrophil count ≥ 1 × 10^9^/l and platelet count > 75 × 10^9^/l. The use of prophylactic granulocyte colony-stimulating factor (G-CSF) was not permitted. Lenalidomide dose was reduced to 5 mg/day if during the preceding cycle neutrophil count fell below 0.5 × 10^9^/l or platelets < 25 × 10^9^/l or if there was a febrile neutropenia (neutrophils < 1 × 10^9^/l).

Serological response was assessed after each induction cycle, before mobilization, at ASCT, 3 months after ASCT and after each cycle during lenalidomide maintenance. Bone marrow (BM) samples were analyzed after induction (RVD × 3) and if a near complete response (nCR, normal electrophoresis but positive immunofixation) or CR was reached. BM MRD was assessed first by multiparameter flow cytometry (MFC) in nCR/CR patients. Allele-specific real-time quantitative polymerase chain reaction (ASO-RQ-PCR) follow-up was activated if stringent CR (sCR)/flow-MRD-negativity was reached. From these patients, BM sample was collected every 3 months during the first year and every 4 months thereafter until at least 2 years on maintenance.

### Multiparameter flow cytometry

The MFC assays were performed in the laboratories of five Finnish university hospitals. BM samples were stained with monoclonal antibodies and analyzed with FacsCanto or FacsCanto II flow cytometers (BD Biosciences) using 6- or 8-color protocols or Navios flow cytometer (Beckman-Coulter) using a 10-color protocol. Quality controls were performed with electronic files and comparison with PCR results. Plasma cells were gated using CD38 and CD138 antibodies and light scatter properties. The expressions of CD45, CD19, CD56, CD27, CD81, CD117, and intracytoplasmic Κ/λ were assessed in all samples at diagnosis and informative markers were used for MFC-MRD analysis. Fifty immunophenotypically abnormal plasma cell events was the lower limit for quantitation in the MRD analysis. The aim was to collect at least 0.5 × 10^6^ total nucleated cells (TNC) to achieve the sensitivity of 0.01%. The median sensitivity of negative samples was < 0.008% (< 0.001 to < 0.06%). The median number of TNC analyzed for MRD-flow was 668,000 (113,000–4,100,000).

### ASO RQ-PCR

The ASO RQ-PCR analyses were centralized in the molecular laboratory of Turku University Hospital. The pre-treatment BM DNA samples were screened for clonal immunoglobulin heavy chain (IgH), immunoglobulin kappa (IgK), and immunoglobulin lambda (IgL) rearrangements using BIOMED-2 multiplex primer sets and heteroduplex analysis. Samples negative with the multiplex primers were further analyzed by additional PCR reactions with family-specific consensus primers. The clonal PCR products were sequenced to determine the variable, diversity, and joining (VDJ)–junction and to design the ASO primers at the junctional region. The ASO primer was first used in RQ-PCR together with the appropriate germline J-gene TaqMan-probe and a reverse primer. In cases with suboptimal assay performance, a patient-specific TaqMan-probe and a reverse-oriented ASO primer were designed and used for MRD analysis [19]. In MRD analyses, the pre-treatment sample served as quantification standard for the follow-up samples. Sensitivity and quantitative range of the analysis as well as the assay setup was determined according to EuroMRD guidelines [20]. The percentage of myeloma cells in the pre-treatment sample as determined by flow cytometry was used for adjusting the final sensitivity. Albumin gene was used for normalization of the ASO RQ-PCR results. 0.5 μg of follow-up sample DNA was used in each of the three replicate analyses. The maximum sensitivity achievable was 4 × 10^−6^ based on the total amount of 1.5 μg of DNA, corresponding to 240,000 nucleated cells. The median sensitivity of PCR-negative samples was < 0.0005% (< 0.0004 to < 0.005%). ASO-PCR assay design was activated for 39 patients and was successful in 34/39 (87%) patients. Due to high rate of somatic mutations, 12/34 (35%) patients needed a patient-specific TaqMan probe.

### Fluorescence in situ hybridization

BM cytogenetics was analyzed by fluorescence in situ hybridization (FISH) and karyotyping in the laboratories of genetics of the participating university hospitals. BM FISH analyses were applied to CD138+ selected cells for IgH (if positive, screening of translocations 4;14, 14;16, 14;20, 11;14), 17 deletion/monosomy, 13 deletion/monosomy, 1q25 gain, 1p36 loss, amplification of chromosome 9, and chromosome 6 aberration.

### Endpoints

The primary endpoints of this study were to determine (1) the rate of flow-MRD-negativity after induction and ASCT including the CR rate and PCR-negativity rate of flow-MRD-negative/sCR patients, (2) improvement of responses during lenalidomide maintenance, and (3) PFS. Secondary endpoints were feasibility of this three-drug induction combination, overall response rate (ORR), number of CD34+ cells collected after low-dose CY + G-CSF vs. G-CSF alone mobilization, number of aphereses, and costs according to the mobilization arm, graft composition, duration of treatment, and OS. The results of the mobilization endpoints [18], graft cellular composition [21], and cost analysis of the mobilization substudy [22] have been reported previously.

### Statistical analysis

The primary efficacy analysis was the intention-to-treat (ITT) and included (*N* = 80) for all the patients who underwent randomization at registration. The safety assessment comprised patients who received any dose of the trial treatment (*N* = 78). Continuous variables were summarized with descriptive statistics and categorical variables were summarized in frequency tables. Pearson’s chi-square test, Fisher’s exact test (no-scale variable difference between different groups), related samples Wilcoxon signed-rank test, related samples McNemar’s test, and Cochran test were used to analyze data.

The data cutoff was 10 April 2018. The PFS was calculated as the time from the inclusion to the first documentation of progressive disease (PD) or death, whichever came first, using competitive risk analysis. The OS was calculated as the time from the inclusion to death or data cutoff. The event-free survival (EFS) was calculated as the time from inclusion to any of the following: PD, death or withdrawal from study for any reason. The Kaplan-Meier method with log-rank test was used to estimate the survival distribution. Competitive risk analysis method was used to analyze, which factors showed independent predictive value for myeloma outcome in multivariate models, using PFS as outcome indicator and withdrawal from study for other reasons than PD or death as a competitive event. Hazard ratios with 95% confidence intervals (CI) and *p* values were reported in all regression models. To test multicollinearity, non-parametric Spearman correlations were analyzed pairwise between all factors, with 2-tailed tests of significance.

All analyses were conducted using IBM SPSS Statistics 22 for PC, IBM Corp. and R (version 3.5.0).

## Results

### Enrolment and patient characteristics

Eighty NDMM patients were enrolled during 28 January 2013–26 February 2015 and randomized to the mobilization arms. Their characteristics are described in Table 1. The median age was 63 (40–70) years. Two patients were withdrawn early (neutropenia, previous cancer) without any study drug administration. Of the 78 patients who started induction, 69 (86% by ITT) were mobilized. Fifty-nine patients (74%) received ASCT, 54 (68%) started lenalidomide maintenance, and 29 (36%) of these are still on maintenance (Online Resource 2).

**Table 1:** Patient characteristics at diagnosis (*N* = 80)

Median age, years (range)	63 (40–70)
Gender, M/F (*N* (%))	42/38 (53/47)
Paraprotein isotype (*N* (%))	
IgG	51 (64)
IgA	16 (20)
Light chain	13 (16)
Hemoglobin (g/L, median (range))	104 (64–141)
P-Creatinine (μmol/L, median (range))	80 (47–404)
S-β2-Microglobulin (mg/L, median (range))	3.1 (1.2–16.6)
Bone marrow plasma cells (% (range))	46 (10–100)
ISS (*N* (%))	
I	21 (26)
II	44 (55)
III	15 (19)
R-ISS (*N* (%))	
I	15 (19)
II	57 (71)
III	8 (10)
IMWG risk	
Low risk	10 (13)
Standard risk	59 (74)
High risk	11 (14)
FISH findings (*N* (%))	
Del13q/-13	29 (36)
Del17p*	7 (9)
+1q	18 (23)
t(4;14)	7 (9)
t(11;14)	8 (10)
t(14;16)	2 (3)
t(6;14)	1(1)
Hyperdiploidy	15 (19)
Other^^^	36 (45)
None	10 (13)

*FISH*, fluorescent in situ hybridization; *IMWG risk*, International Myeloma Working Group Risk Stratification; *ISS*, International Staging System; *R-ISS*, Revised International Staging System

*Del17p proportion, median 85% (11–94%), one patient had 11% and all the others > 60%

^^^del1p32/1p36, del2, -6, del6q, del10, del14q, -12, -16, del16q, t8;14, add14q32, 14q translocations

### Primary endpoints

#### The rate of flow-MRD-negativity and PCR-negativity

The ORR (≥ PR) was 89% including sCR rate of 38%, CR 10%, VGPR 20%, and PR 21%. Flow-MRD-negativity at least once independent of serological response was achieved in 53% (42/80) of the patients. After induction, 29% (23/80) were flow-MRD negative, after ASCT 35%, at 1 year 26%, at 2 years 23%, and at 3 years after ASCT 15% (Table 2). Sustained flow-MRD-negativity for ≥ 1 year was reached in 29% (23/80) of the patients. PCR-negativity was reached in 28% (22/80) of the patients and 11% (9/80) had sustained PCR-negativity for ≥ 1 year. There was no difference in serological, immunophenotypic, or molecular response rates according to the randomization arm. The median time to response (PR or better) was 22 (14–202) days and the median time to the best serological response achieved during study 4.5 (0–45) months. There was no difference in time to response between patients with HR cytogenetics and those without them. The HR cytogenetic group (20% [16/80]) was defined here by the presence of del17p (any percentage), t(4;14), or t(14;16). Of these HR patients, 44% reached sCR compared with 36% of the non-HR patients (*p* = 0.71).

**Table 2:** Summary of treatment responses in the whole study population (intention to treat, *N* = 80)

Response	After induction therapy	At 3 months after ASCT	Len maintenance 1 year after ASCT	Len maintenance 2 years after ASCT	Len maintenance 3 years after ASCT	Best response at any time+
sCR (*N* (%))	8 (10)	12 (15)	13 (16)	16 (20)	16 (20)	30 (38)
CR (*N* (%))	4 (5)	6 (8)	9 (11)	7 (9)	5 (6)	8 (10)
VGPR (*N* (%))	28 (35)	25 (31)	14 (18)	10 (13)	7 (9)	16 (20)
PR (*N* (%))	28 (35)	10 (13)	5 (6)	1 (1)	1 (1)	17 (21)
SD (*N* (%))	2 (3)	1 (1)	0	0	0	1 (1)
Cumulative PD (*N* (%))	1 (1)	11 (14)	20 (25)	26 (33)	30 (38)	-
Flow-MRD negative^^^ (*N* (%))	23 (29)	28 (35)	21 (26)	18 (23)	12 (15)	42 (53)
PCR-MRD negative^^^ (*N* (%))	4 (5)	8 (10)	7 (9)	8 (10)	3 (4)	22 (28)
Cumulative withdrawn* (*N* (%))	9 (11)	15 (19)	19 (24)	20 (25)	21 (26)	-

*ASCT*, autologous stem cell transplantation; *CR*, complete response; *Len*, lenalidomide; MRD, minimal residual disease; *nCR*, near complete response; *PD*, progressive disease; *PR*, partial response; *sCR*, stringent complete response; *SD*, stable disease; *VGPR*, very good partial response

^^^Regardless of serological response

*Withdrawn at induction phase: 1 with other cancer < 5 years, 1 with neutropenia; at mobilization or ASCT before lenalidomide maintenance start, 1 with simultaneous other cancer, 1 with thrombosis, 1 not eligible to ASCT, 1 death, 1 liver toxicity, 1 infection toxicity, 1 with severe sepsis syndrome, 1 with comorbidities, 3 received allogeneic SCT, 1 psychiatric illness, 1 rash +response could not be evaluated due to early withdrawal for 7 patients and one patient progressed early during induction

#### Improvement of responses during maintenance

Among the patients who were not progressed or withdrawn at the start of maintenance (*N* = 54), the paraprotein responses improved during the first and second year after ASCT (Table 2). This improvement was statistically significant between the start of maintenance and 1 year (*p* = 0.01, related samples Wilcoxon signed-rank test) and between 1 and 2 years after ASCT (*p* = 0.03). At the start of maintenance therapy and at 1 and 2 years after ASCT, sCR rates were 22% (12/54), 24% (13/54), and 30% (16/54), respectively. Flow-MRD-negativity, regardless of paraprotein response, was achieved in 67% (36/54) of these patients at least once and 37% (20/54) reached also PCR-negativity once. The changes in flow- or PCR-negativity rates during maintenance therapy were not statistically significant.

Of the 29 patients who are still on lenalidomide maintenance at the cutoff point, 17/29 (59%) are in sCR, 4 (14%) in CR, 7 (24%) in VGPR, and 1 (3%) in PR. The paraprotein response of these long-term responders deepened significantly during the first (*p* = 0.004) and the second (*p* = 0.011) year after ASCT on maintenance but not during the third year (*p* = 0.180). The proportion of flow-MRD-negative patients increased during the first year of maintenance, from 59 (17/29) to 72% (21/29), *p* = 0.046, but not thereafter. PCR-negativity rate did not change significantly during maintenance in this patient group.

Of the 29 patients who are still on maintenance, 24% (7/29) have hyperdiploidy; 28% (8/29) normal FISH; 10% (3/29) t(11;14); 17% (5/29) del13, 2 with +1q; 17% (5/29) +1q alone, del1p or undefined IgH translocation, and only one (3%) del17. Of these 29 patients, 19 (66%) have sustained flow-MRD-negativity and 11/19 (58%) have either hyperdiploidy, t(11;14), or no FISH findings. Twenty-one percent (6/19) have sustained PCR-negativity.

#### Survival outcomes

**Fig. 1:**
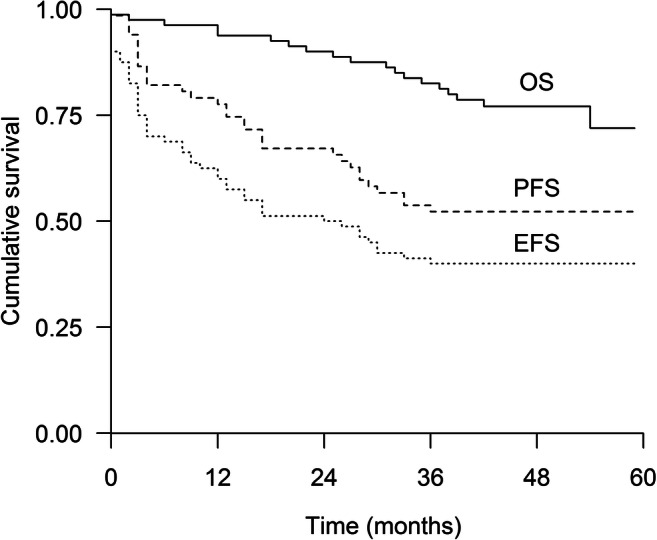
Progression-free survival (PFS), event-free survival (EFS), and overall survival (OS) of the study patients (*N* = 80)

With a median follow-up of 27 (0–59) months, neither median PFS nor OS have been reached. The PFS at 1, 2, and 3 years after inclusion was 78%, 67%, and 52%, and OS 96%, 90%, and 83%, respectively. Median EFS was 24 months (95% CI 9–39 months) (Fig. 1). The median PFS for the Revised Multiple Myeloma International Staging System (R-ISS) stage 3 group was only eight (95% CI 0–27) months and was not reached for the other groups. For the patients who started maintenance (*N* = 54), PFS at 1, 2, and 3 years after inclusion was 94%, 80%, and 65% and OS 100%, 96%, and 87%, respectively.

**Fig. 2:**
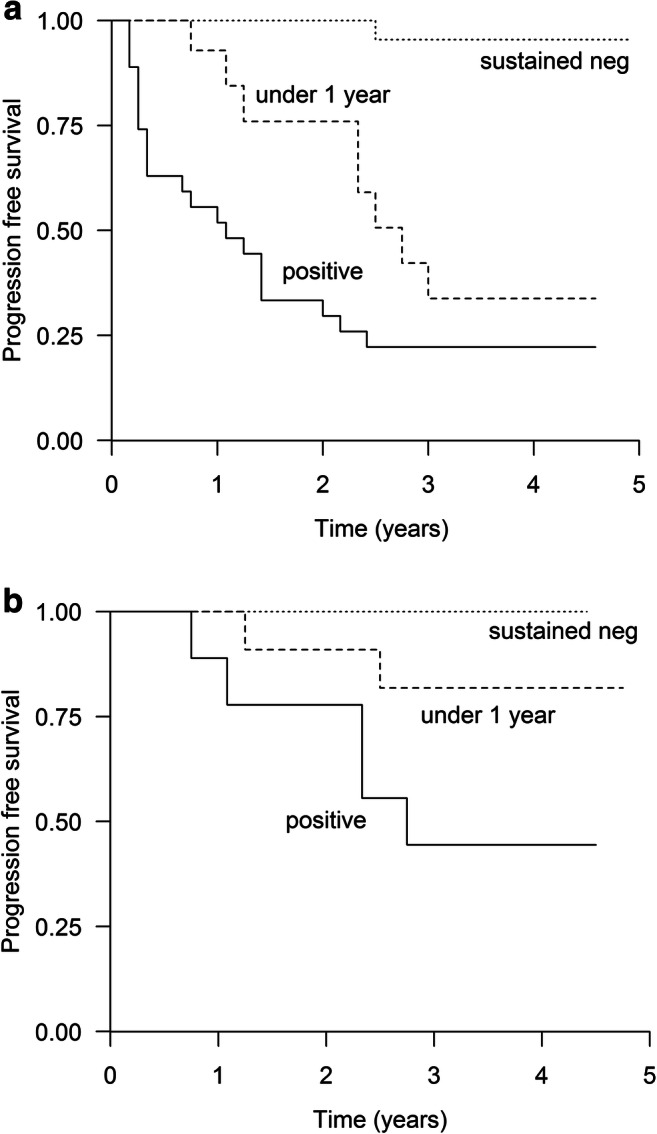
Progression-free survival **a** according to the flow-MRD status *N* = 69, positive *N* = 27; negative for < 1 year *N* = 19; sustained negative *N* = 23, *p* < 0.001. **b** According to the PCR-status *N* = 32, positive *N* = 10; negative for < 1 year *N* = 13; sustained negative *N* = 9, *p* = 0.044

If flow-MRD-negativity was achieved and sustained for at least 1 year, the median PFS was not reached compared with the patients who achieved flow-MRD-negativity but lost it below 1 year (median PFS 33 months; 95% CI 27–39) and to the MRD-flow-positive patients (median PFS 15 months; 95% CI 9.9–20.1), *p* < 0.001 (Fig. 2a). The median OS was not reached for any of these groups (*N* = 69, *p* = 0.01).

Of the patients who achieved PCR-negativity for at least 1 year, 11% (9/80), none have relapsed to date. The median PFS (*p* = 0.044) (Fig. 2b) or OS (*p* = 0.443) was not reached either in this group or groups with PCR-negativity less than 1 year or PCR-positive.

No statistically significant differences were seen in terms of IMWG, R-ISS, ISS, age, gender, or study arm between sustained flow-MRD-negativity group and others or between sustained PCR-negativity group and others, but the number of PCR-negative patients was small. Nine patients have reached sustained PCR-negativity and only one of them had HR cytogenetics.

#### Duration of the treatment

The median follow-up of all patients (*N* = 80) is 27 (0–59) months and for those patients (*N* = 54) who started maintenance 43 (8–59) months. The median duration of lenalidomide maintenance therapy is so far 33 (0–51) months.

To date, 29 of the total of 80 patients (36% by ITT) are still on maintenance treatment. Of those 54/80 patients who started maintenance, 25 (46%) have discontinued it, 19/54 (35%) due to PD, four due to side effects, and two by their own will. Five (6% by ITT) patients who received ASCT did not start maintenance at all: two due to PD, one due to severe rash during induction, one by investigator’s decision, and one proceeded to allogeneic stem cell transplantation.

**Fig. 3:**
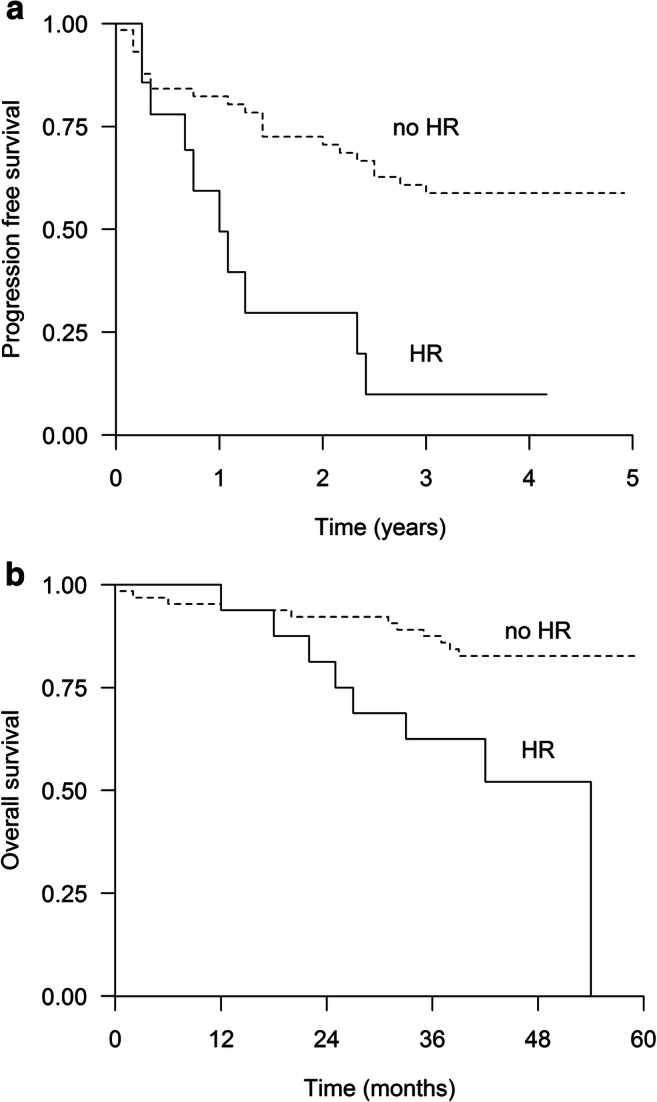
**a** Progression-free survival (*p* < 0.01) and **b** overall survival (*p* = 0.003) for patients with high-risk (HR) cytogenetics (*N* = 16) and no high risk (no HR) (= 64)

The median PFS and OS for the HR patients were only 15 (95% CI 5–25) and 54 (12–54) months, respectively. In the no-HR group, PFS (Fig. 3a) or OS (Fig. 3b) were not reached. The estimated median EFS for HR and no-HR patients was eight (95% CI 0.4–16) months vs. not reached, *p* < 0.001. Only one HR patient achieved sustained flow-MRD-negativity, six were in non-sustained group, and six in the MFC-positive group (*p* = 0.079). In the HR group, 11 patients (69%) have relapsed to date compared with 25 (39%) in the non-HR group (*p* = 0.033). Four HR patients have withdrawn from the study. Eight HR patients started lenalidomide maintenance but seven of them have relapsed after a median of only 6 months on maintenance.

To test the predictive impact of different variables on PFS, gender, age ≤ or > 65 years, study arm, World Health Organization (WHO) performance status, IMWG group, R-ISS, ISS, HR cytogenetics, best serological response (sCR vs. CR/VGPR vs. PR/worse), and achievement of flow-MRD-negativity or PCR-negativity were tested with multivariate analyses. In correlation analysis, there proved to be high pairwise correlations, between 0.677 and 0.773 between flow-MRD-negativity, PCR-negativity, and serological responses, and due to this multicollinearity, these three factors competed for the same predictive power. In multivariate analysis, only one of them, achievement of flow-MRD-negativity, provided independent prognostic significance for PFS (hazard ratio 4.31, 95% CI 2.08–8.92, *p* < 0.001) (Online Resource 1).

### Secondary endpoint: adverse events

Adverse events (AEs) were assessed from all the patients (*N* = 78) who received at least one dose of trial treatment. Lenalidomide maintenance AEs were separately assessed from the patients (*N* = 54) who received at least one dose on maintenance therapy.

AEs grade 3 or higher are reported in Table 3. Hematological and infectious AEs were the most common. During RVD induction, 17 (22%) febrile neutropenic events were reported. The rate of severe peripheral neuropathy was low, only 3%. Treatment-related mortality for the whole protocol was 1%. During induction, one patient died due to hepatorenal syndrome caused most probably by bortezomib (autopsy).

**Table 3:** Grade ≥ 3 adverse events in the treated population

Adverse event	RVD induction *N* = 78 (%)	ASCT *N* = 59 (%)	Lenalidomide maintenance *N* = 54 (%)
Hematologic adverse events			
Neutropenia	19 (24)	58 (98)	34 (63)
Febrile neutropenia	17 (22)	46 (78)	11 (20)
Thrombocytopenia	11 (14)	53 (90)	3 (6)
Anemia	6 (8)	15 (25)	0
Infections*	18 (23)		8 (15)
Gastrointestinal disorders	5(6)	3 (5)	0
Diarrhea	3 (4)	3 (5)	0
Hepatobiliary disorders	4^^^ (5)	0	1 (2)
Nervous system disorders	2 (3)	2 (3)	1 (2)^e^
Peripheral neuropathy	2 (3)	2 (3)	0
Skin disorders			
Urticaria/rash	5 (6)	1 (2)	3 (6)
Dry skin	0	0	1 (2)
Thromboembolic event	3 (4)	2 (3)	1 (2)
Cardiac disorders	2 (3)	1 (2)	1 (2)
SPMs^+^	0	0	2 (4)
Other^#^	4 (5)	0	2 (4)

*AE*, adverse event; *ASCT*, autologous stem cell transplantation; *RVD*, lenalidomide, bortezomib, and dexamethasone; *SPM*, secondary primary malignancy

*Febrile neutropenias not included since reported separately

^One grade 5 adverse event, hepatorenal syndrome resulting in death

^e^One ADEM (acute disseminated encephalomyelitis)

^+^Basalioma during Len maintenance, squamocellular carcinoma of tonsilla, diagnosed 1 year after discontinuation of lenalidomide

^#^During induction: bone pain 2 (3%), syncope 1 (1%), dyspnea 1 (1%), during maintenance interstitial pneumonia 1 (1%), pneumothorax 1 (1%)

During the lenalidomide maintenance, 28 (52%) patients had grade 3 neutropenia, 6 (11%) grade 4 neutropenia, and 11 (20%) had febrile neutropenia. Lenalidomide dose was reduced from 10 to 5 mg per day in 16/54 (30%) patients. Reasons for dose reductions were neutropenia in ten (63%), neutropenic pneumonia in one, rash/urticaria in three, grade 3 thrombocytopenia in one, and vertigo and cephalalgia in one patient.

Maintenance treatment was permanently discontinued due to side effects in four patients: two for rash, one for interstitial pneumonia, and one for SPM (basalioma). These patients are in the follow-up and three of them have not progressed within the follow-up.

Two SPMs (2.5%) have been reported so far. One patient had facial basalioma during maintenance and one patient had previously discontinued maintenance due to the rash after 2 months of use, and 1 year later, grade 3 squamocellular carcinoma of the left tonsilla was diagnosed. This patient is in remission after chemoradiotherapy.

## Discussion

The main aim of this FMG-MM02 study was to evaluate the flow-MRD-negativity rate after RVD induction followed by randomized stem cell mobilization phase, single ASCT, and lenalidomide maintenance. Lenalidomide was not approved for induction in transplant-eligible patients at the study start. Maintenance after ASCT is now an indication for lenalidomide use but not yet reimbursed in Finland.

The early treatment phase may be pivotal for a new MM patient because it can be compromised by excessive toxicity of the induction regimen. In this small study, seven patients (9%) dropped out early due to toxicities (Online Resource 2), followed by treatment delay and early MM progression in some of them. RVD combination is one of the recommended 3-drug induction treatments before ASCT [1, 23, 24]. In the French RVD trial, the incidence of ≥ grade 3 infections was 6%, and 35% of patients had ≥ grade 3 neutropenia [23]. In IFM2009 trial, the respective numbers were 8.9% and 47.4% [25], and in SWOG S0777, 15% and 47% [26]. The proportion of ≥ grade 3 infections is not less in other recommended 3-drug inductions: 26%, 21%, 22% in bortezomib, doxorubicin, and dexamethasone (PAD); bortezomib, thalidomide, and dexamethasone (VTD); and bortezomib, cyclophosphamide, and dexamethasone (VCD) combination, respectively, and 15% with carfilzomib, lenalidomide, and dexamethasone (KRD) [24]. In our trial, 35 (45%) serious infectious events were reported, half of them in neutropenic patients despite the lenalidomide duration of only 14 days (Table 3).

The Spanish group has recently published their results of the PETHEMA/GEM2012 study [27] with 6 cycles of RVD (28-day regimen) followed by ASCT and RVD consolidation in NDMM patients. In their study, CR was achieved by 33% after 6-course induction and 44% after ASCT in ITT population compared with 15% after 3 courses of RVD and 23% after ASCT in our trial, respectively, but the differences in cycle length and the number of induction cycles must be considered. The responses after 3 cycles in ITT population in the PETHEMA/GEM2012 study were not reported. Patients in the PETHEMA/GEM2012 study were younger (median age 58 years) than in Finland (63 years). The proportion of high-risk cytogenetics patients by del17p, t(4;14), and/or t(14;16) was the same in both studies (20%). The proportion of ISS stage II patients was higher in our trial, 55% vs. 36.2% in the Spanish trial. The rate of ≥ grade 3 infections and peripheral neuropathy during induction were 9.2% and 3.9% in the Spanish trial compared with 23% and 3% after 3 cycles in our study, respectively. A longer induction phase may be an approach worth further investigation.

The approved maintenance dose of lenalidomide by EMA is 10 mg daily continuously, given until disease progression or intolerance. After three cycles of lenalidomide maintenance, the dose can be increased to 15 mg daily if tolerated (https://www.ema.europa.eu/documents/product-information/revlimid-epar-product-information_en.pdf). Based on the meta-analysis of three lenalidomide maintenance trials, grades 3–4 neutropenia were quite common (23–51%) and rate of infections 6–13% [5]. The lowest neutropenia rate, 23%, was in the trial of Palumbo et al. [4], where the dose was 10 mg daily on days 1–21 in a 28-day cycle. In the RVD study [23], the maintenance dose was 10 mg daily, escalating to 15 mg daily at 3 months according to blood cell counts and safety. Only 37% of the patients received lenalidomide maintenance at the planned full dose in that trial, myelosuppression with ≥ grade 3 neutropenia being the most common limiting factor. The rate of grades 3–4 neutropenia was 60% during maintenance and 63% of patients had dose reductions because of AEs, mostly neutropenia. Finally, permanent discontinuation of lenalidomide maintenance occurred in 29.1% of patients by meta-analysis [5].

In our study, lenalidomide maintenance even at the dose of 10 mg daily on 21 days in 28-day cycles was not well tolerated. Reduction to 5 mg daily dose was needed in 30% of patients, 63% had ≥ grade 3 neutropenia, and one-third of them had febrile neutropenia. The permanent discontinuation rate was still lower, 11%, than reported earlier [5]. With this background, the recommended dose escalation to 15 mg daily continuously might be challenging. In non-high-risk patients, the continuity of maintenance, not the dose, might be the most important contributor for favorable outcome. Prophylactic G-CSF use was not permitted in our study but EMA accepts its use at the physician’s discretion (https://www.ema.europa.eu/documents/product-information/ revlimid-epar-product-information_en.pdf). However, the other potential maintenance drugs are not without AEs either; bortezomib caused infections in 24% of patients without any neutropenia, and for thalidomide, the infection rate was 18% with the neutropenia rate of 1–16% [24, 28].

In the French trial, patients received lenalidomide during RVD cycles on days 1–21 also as consolidation and lenalidomide maintenance was limited to 1 year [23]. The mere goal of flow-MRD-negativity at any time point irrespective of other response criteria was reached in 68% of the patients in the French trial compared with 53% observed in our study. Our flow-panel included CD27/CD81/CD117 in addition to those antigens used in the French panel and the median sensitivity of the assay was 10^−4^. By using this MFC analysis, the flow-MRD-negativity correlated with prolonged PFS and identified the patients with sustained flow-MRD-negativity and long PFS. None of the patients with sustained PCR-negativity, the method displaying one logarithm higher sensitivity than that of the flow, has relapsed so far. The 3-year OS is 83% compared with 100% in the French trial. Forty-six percent (6/13) of the patients in our trial with an early death had HR cytogenetics and two of them died despite an allogeneic SCT after withdrawal from trial.

Minimal residual disease is a powerful prognostic marker in MM [9–14]. In the present study, MRD was detected by conventional 6–10-color MFC and samples without detectable MRD by flow were further tested by quantitative patient-specific ASO-PCR. In our study, the median sensitivity of the flow-MRD analysis was 0.01%. Achieving flow-MRD-negativity even with this conventional MFC predicted longer PFS. Of note, higher sensitivity of MRD detection was achieved by applying ASO-PCR analysis for flow-negative samples. The median sensitivity of PCR-negative samples was 0.0005%. ASO-PCR assay design was not successful in 5 (13%) patients due to lack of clonal Ig rearrangement in the pre-treatment sample. This was most probably due to somatic hypermutations in the primer-binding regions or too low a fraction of myeloma cells in the sample taken for molecular genetic analyses. Recently, high-sensitivity MFC methods (NGF, next-generation flow cytometry) with detection limit of 0.001% or even 0.0001% have been developed [29]. The clinical studies applying this technique have confirmed that even very low level of MRD has negative impact on patient outcome [30]. Similarly, more sensitive and more widely applicable genetic methods, like next-generation sequencing (NGS) and digital-droplet PCR, have been introduced for clinical studies [31–33].

The kinetics of response during lenalidomide maintenance showed deepening of serological responses during the first 2 years of maintenance but only a trend for increasing the rate of flow-MRD-negativity was found during the first year of maintenance. The use of maintenance until PD is approved but to give it continuously for all patients or until CR or MRD negativity needs to be investigated more. In the study by Goldschmidt et al. [7], where patients were randomized to receive lenalidomide maintenance for 2 years or until achievement of CR, toxicity was increased in the groups receiving lenalidomide for 2 years. Grade ≥ 2 infections were the major AE during maintenance.

Considering stem cell mobilization, CY + G-CSF was more effective compared with G-CSF alone without marked difference in toxicity but also G-CSF alone was successful in a great majority of patients to reach the defined collection target, and in all patients, the minimum collection target was achieved. In the light of these results, mobilization with G-CSF alone could be considered at least in patients who are intended for single autograft.

Limitations of this study are the non-randomized design in addition to the limited number of patients. Accordingly, we do not know whether the outcome of the patients would have been similar without the maintenance. However, this is to our best knowledge the only study including comprehensive cytogenetic data of the patients. In addition, all the patients in this study achieving ≥ nCR/CR were followed by sequential MRD assessments for at least 2 years during the maintenance.

In conclusion, we noticed flow-MRD-negativity rate with the sensitivity of 10^−4^ in 53% of patients using conventional MFC and PCR-negativity rate of 10^−5^ in 28% of patients during a short RVD induction, single ASCT, and lenalidomide maintenance. Achieving flow-MRD-negativity even with this conventional MFC predicted longer PFS. During maintenance, treatment responses improved mostly during the first 2 years. The outcome of HR patients was poor with this protocol treatment. To reach a more favorable long-term outcome, they need adjusted prolonged treatment combining immunomodulatory agents with proteasome inhibitors and probably with monoclonal antibodies and avoidance of any interruption of therapy due to excessive toxicity.

## Electronic supplementary material

ESM 1 (PDF 174 kb)

ESM 2 (PDF 109 kb)

ESM 3 (PDF 131 kb)

ESM 4 (PDF 2066 kb)

ESM 5 (PDF 237 kb)

## Data Availability

The datasets generated during and/or analyzed during the current study are available from the corresponding author on reasonable request.
