# Supplementary material for: RVD induction and autologous stem cell transplantation followed by lenalidomide maintenance in newly diagnosed multiple myeloma: a phase 2 study of the Finnish Myeloma Group
Source: Ann Hematol. 2019 Oct 31;98(12):2781–92. doi: 10.1007/s00277-019-03815-7 (PMC6900265; doi:10.1007/s00277-019-03815-7)
Supplement: Supplementary file 1 — (PDF 174 kb) [file 277_2019_3815_MOESM1_ESM.pdf]

ESM 1 Univariate and multivariate analysis using progression-free survival as an outcome indicator

Variable	Univariate hazard ratio	Univariate *p* value	Multivariate after elimination of serological response, hazard ratio	Multivariate after elimination of serological response, *p* value	Multi-variate, last step, hazard ratio	Multivariate, last step, *p* value
Female vs male	1.09 (0.55 – 2.17)	0.800				
Age > 65 vs ≤ 65 years	0.69 (0.32 – 1.49)	0.340				
Study arm: mobilization with G-CSF alone vs cyclophosphamide + G-CSF	1.22 (0.61 – 2.41)	0.580				
WHO performance status	0.61 (0.33 – 1.12)	0.110				
IMWG group						
-Standard risk vs low risk	1.22 (0.38 – 3.92)	0.740				
-High risk vs low risk	2.75 (0.77 – 9.83)	0.120				
R-ISS						
-2 vs 1	1.25 (0.80 – 3.20)	0.640				
-3 vs 1	1.43 (0.70 – 6.61)	0.650				
ISS						
-2 vs 1	0.90 (0.39 – 2.10)	0.810				
-3 vs 1	1.31 (0.47 – 3.69)	0.610				
High risk vs no high risk cytogenetics	2.49 (1.19 – 5.21)	0.016	2.02 (0.91 – 4.48)	0.084		
Best serological response						
-CR or VGPR vs sCR	2.96 (1.09 – 8.04)	0.033				
-PR or worse vs sCR	6.76 (2.46 – 18.54)	<0.001				
Not achieving flow-MRD negativity vs achieving	4.31 (2.08 – 8.92)	<0.001	2.5 (1.15 – 5.44)	0.021	4.31 (2.08 – 8.92)	<0.001
Not achieving PCR-negativity vs achieving	7.77 (1.94 – 31.1)	0.004	3.87 (0.86 – 17.41)	0.078		

ASCT, autologous stem cell transplantation; CR, complete response; G-CSF, granulocyte colony stimulating factor; IMWG, International Myeloma Working Group; ISS, International Staging System; MRD, minimal residual disease; PR, partial response; R-ISS, Revised International Staging System; sCR, stringent complete response; VGPR, very good partial response; WHO, World Health Organization

Article title: RVD induction and autologous stem cell transplantation followed by lenalidomide maintenance in newly diagnosed multiple myeloma: a phase 2 study of the Finnish Myeloma Group

Journal: Annals of Hematology

Authors: Sini Luoma, Pekka Anttila, Marjaana Säily, Tuija Lundan, Jouni Heiskanen, Timo Siitonen, Sakari Kakko, Mervi Putkonen, Hanna Ollikainen, Venla Terävä, Marja Sankelo, Anu Partanen, Kirsi Launonen, Anu Räsänen, Anu Sikiö, Merja Suominen, Piotr Bazia, Kristiina Kananen, Juha Lievonen, Tuomas Selander, Tarja-Terttu Pelliniemi, Sorella Ilveskero, Virva Huotari, Pentti Mäntymaa, Anri Tienhaara, Esa Jantunen, Raija Silvennoinen

Corresponding author: Sini Luoma, M.D. Comprehensive Cancer Center, Department of Hematology, Helsinki University Hospital and University of Helsinki, Helsinki, Finland. sini.luoma@hus.fi
