# Supplementary material for: RVD induction and autologous stem cell transplantation followed by lenalidomide maintenance in newly diagnosed multiple myeloma: a phase 2 study of the Finnish Myeloma Group
Source: Ann Hematol. 2019 Oct 31;98(12):2781–92. doi: 10.1007/s00277-019-03815-7 (PMC6900265; doi:10.1007/s00277-019-03815-7)
Supplement: Supplementary file 2 — (PDF 109 kb) [file 277_2019_3815_MOESM2_ESM.pdf]

ESM 2 Flowchart for patients in FMG MM02 study according to the randomization arm

Abbreviations: ASCT, autologous stem cell transplantation, PD, progressive disease, RVD, lenalidomide-bortezomib-dexamethasone, SCT, stem cell transplantation.

Article title: RVD induction and autologous stem cell transplantation followed by lenalidomide maintenance in newly diagnosed multiple myeloma: a phase 2 study of the Finnish Myeloma Group

Journal: Annals of Hematology

Authors: Sini Luoma, Pekka Anttila, Marjaana Säily, Tuija Lundan, Jouni Heiskanen, Timo Siitonen, Sakari Kakko, Mervi Putkonen, Hanna Ollikainen, Venla Terävä, Marja Sankelo, Anu Partanen, Kirsi Launonen, Anu Räsänen, Anu Sikiö, Merja Suominen, Piotr Bazia, Kristiina Kananen, Juha Lievonen, Tuomas Selander, Tarja-Terttu Pelliniemi, Sorella Ilveskero, Virva Huotari, Pentti Mäntymaa, Anri Tienhaara, Esa Jantunen, Raija Silvennoinen

Corresponding author: Sini Luoma, M.D. Comprehensive Cancer Center, Department of Hematology, Helsinki University Hospital and University of Helsinki, Helsinki, Finland. sini.luoma@hus.fi

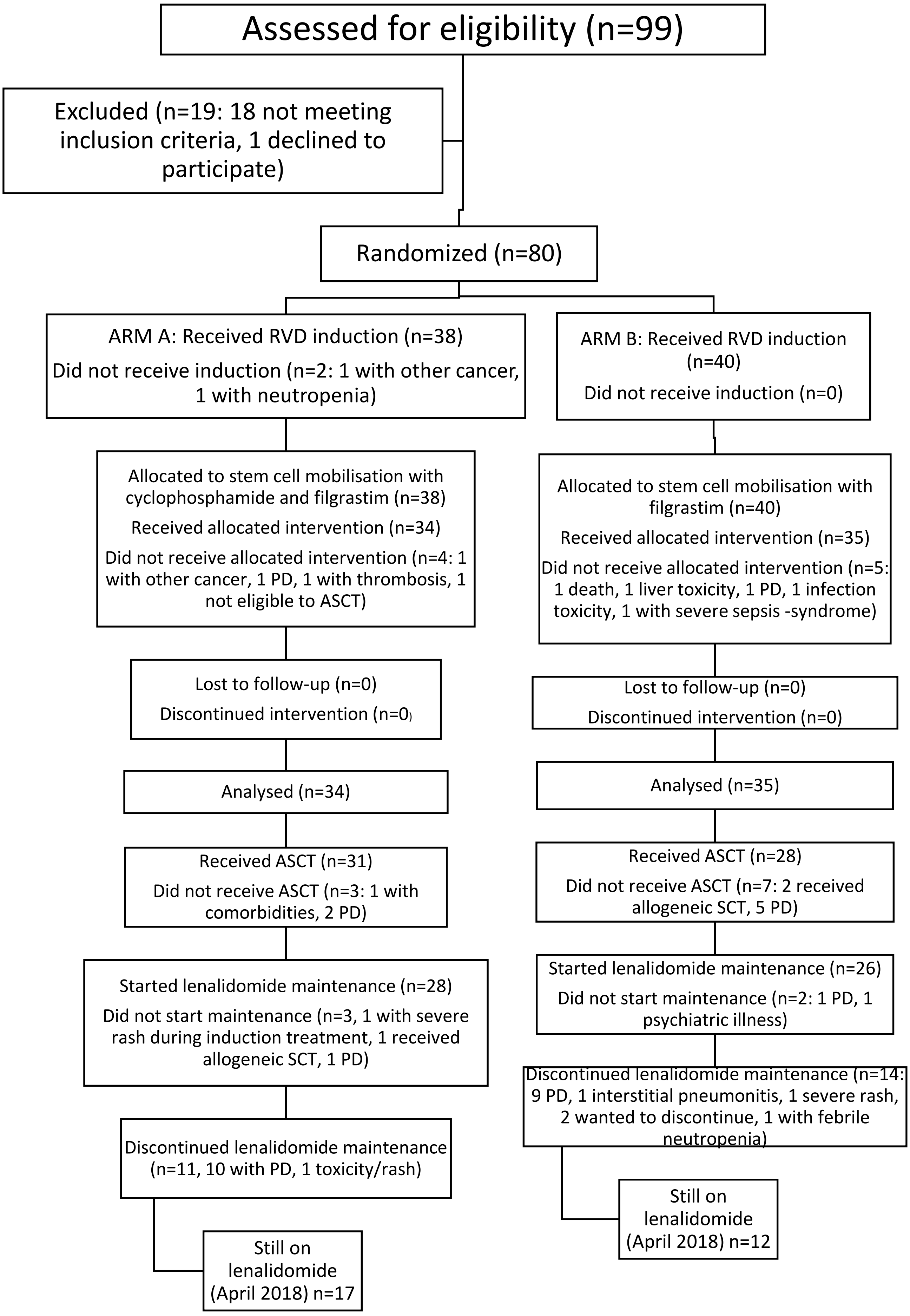
Assessed for eligibility (n=99)
Excluded (n=19: 18 not meeting inclusion criteria, 1 declined to participate)
Randomized (n=80)
ARM A: Received RVD induction (n=38)
Did not receive induction (n=2: 1 with other cancer, 1 with neutropenia)
ARM B: Received RVD induction (n=40)
Did not receive induction (n=0)
Allocated to stem cell mobilisation with cyclophosphamide and filgrastim (n=38)
Received allocated intervention (n=34)
Did not receive allocated intervention (n=4: 1 with other cancer, 1 PD, 1 with thrombosis, 1 not eligible to ASCT)
Allocated to stem cell mobilisation with filgrastim (n=40)
Received allocated intervention (n=35)
Did not receive allocated intervention (n=5: 1 death, 1 liver toxicity, 1 PD, 1 infection toxicity, 1 with severe sepsis -syndrome)
Lost to follow-up (n=0)
Discontinued intervention (n=0)
Lost to follow-up (n=0)
Discontinued intervention (n=0)
Analysed (n=34)
Analysed (n=35)
Received ASCT (n=31)
Did not receive ASCT (n=3: 1 with comorbidities, 2 PD)
Received ASCT (n=28)
Did not receive ASCT (n=7: 2 received allogeneic SCT, 5 PD)
Started lenalidomide maintenance (n=28)
Did not start maintenance (n=3, 1 with severe rash during induction treatment, 1 received allogeneic SCT, 1 PD)
Started lenalidomide maintenance (n=26)
Did not start maintenance (n=2: 1 PD, 1 psychiatric illness)
Discontinued lenalidomide maintenance (n=11, 10 with PD, 1 toxicity/rash)
Discontinued lenalidomide maintenance (n=14: 9 PD, 1 interstitial pneumonitis, 1 severe rash, 2 wanted to discontinue, 1 with febrile neutropenia)
Still on lenalidomide (April 2018) n=17
Still on lenalidomide (April 2018) n=12
