# Supplementary material for: RVD induction and autologous stem cell transplantation followed by lenalidomide maintenance in newly diagnosed multiple myeloma: a phase 2 study of the Finnish Myeloma Group
Source: Ann Hematol. 2019 Oct 31;98(12):2781–92. doi: 10.1007/s00277-019-03815-7 (PMC6900265; doi:10.1007/s00277-019-03815-7)
Supplement: Supplementary file 3 — (PDF 131 kb) [file 277_2019_3815_MOESM3_ESM.pdf]

ESM 3 FMG MM02 study design

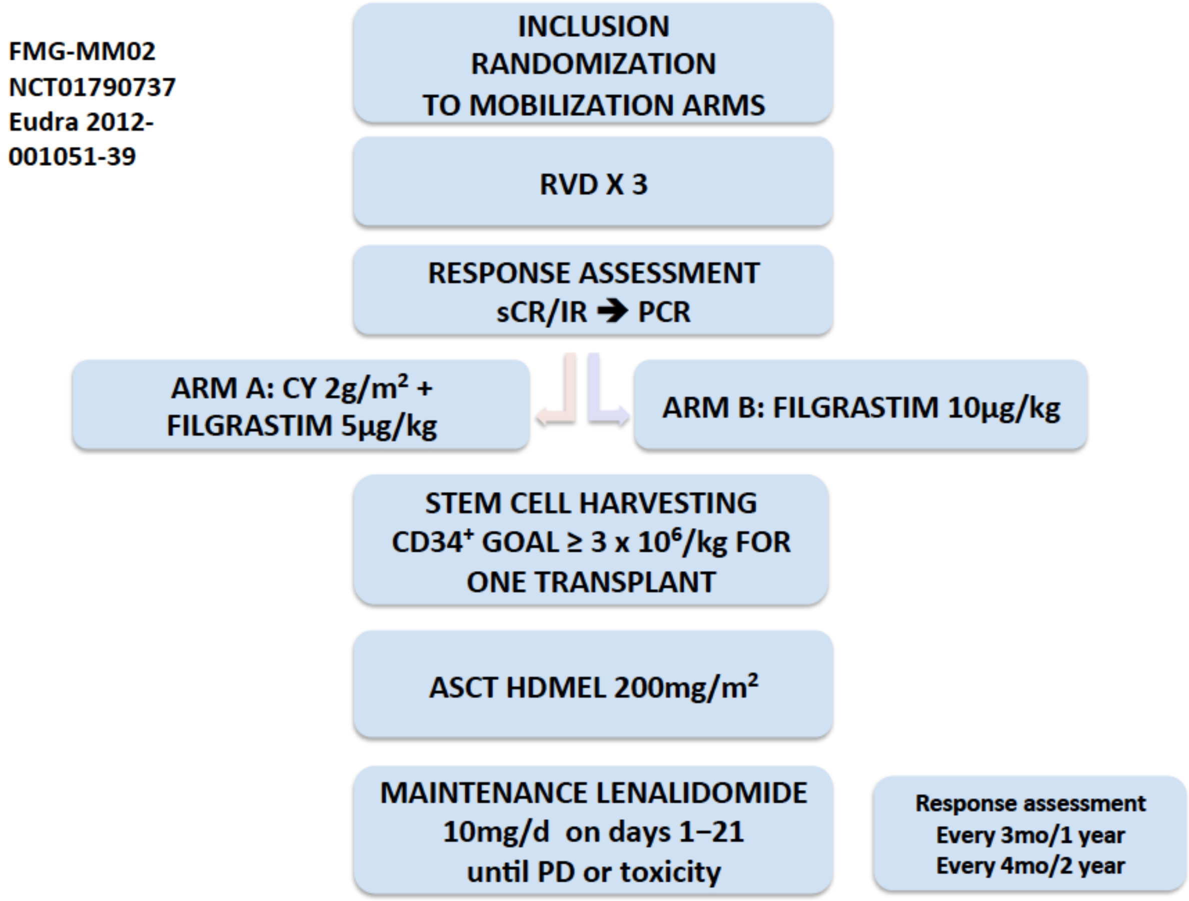

Abbreviations: RVD, lenalidomide-bortezomib-dexamethasone; ASCT, autologous stem cell transplantation; sCR, stringent complete response; IR, immunophenotypic remission (flow-MRD negativity); PCR, polymerase chain reaction; CY, cyclophosphamide; HDMEL, melphalan 200 mg/m2; PD, progressive disease

Article title: RVD induction and autologous stem cell transplantation followed by lenalidomide maintenance in newly diagnosed multiple myeloma: a phase 2 study of the Finnish Myeloma Group

Journal: Annals of Hematology

Authors: Sini Luoma, Pekka Anttila, Marjaana Säily, Tuija Lundan, Jouni Heiskanen, Timo Siitonen, Sakari Kakko, Mervi Putkonen, Hanna Ollikainen, Venla Terävä, Marja Sankelo, Anu Partanen, Kirsi Launonen, Anu Räsänen, Anu Sikiö, Merja Suominen, Piotr Bazia, Kristiina Kananen, Juha Lievonen, Tuomas Selander, Tarja-Terttu Pelliniemi, Sorella Ilveskero, Virva Huotari, Pentti Mäntymaa, Anri Tienhaara, Esa Jantunen, Raija Silvennoinen

Corresponding author: Sini Luoma, M.D. Comprehensive Cancer Center, Department of Hematology, Helsinki University Hospital and University of Helsinki, Helsinki, Finland. sini.luoma@hus.fi
