# Supplementary material for: RVD induction and autologous stem cell transplantation followed by lenalidomide maintenance in newly diagnosed multiple myeloma: a phase 2 study of the Finnish Myeloma Group
Source: Ann Hematol. 2019 Oct 31;98(12):2781–92. doi: 10.1007/s00277-019-03815-7 (PMC6900265; doi:10.1007/s00277-019-03815-7)
Supplement: Supplementary file 4 — (PDF 2066 kb) [file 277_2019_3815_MOESM4_ESM.pdf]

“A prospective phase II study to assess immunophenotypic remission after three-drug induction followed by randomized stem cell mobilization, autologous stem cell transplantation and lenalidomide maintenance in patients with newly diagnosed multiple myeloma”

Confidential

**EudraCT number**	2012-001051-39	
**Protocol number**	FMG-MM-02	
**Final version 1.0**	Date: 7.6.2012	
**Sponsor**	Kuopio University Hospital,<br>Clinic of Internal Medicine/Center of Medicine	
**Coordinating Investigator**	Raija Silvennoinen	Kuopio University Hospital
**Principal Investigator’s**	Pekka Anttila	Helsinki University Hospital
	Tuomo Honkonen	Lahti City Hospital
	Kristiina Kananen	Kainuu Central Hospital
	Anu Kutila	Mikkeli Central Hospital
	Kirsi Launonen	Länsi-Pohja Central Hospital
	Päivi Lehtonen	Savonlinna Central Hospital
	Anne Nihtinen	Joensuu Central Hospital
	Minna Nyypyy	Kokkola Hospital
	Jorma Opas	Seinäjoki Central Hospital
	Eira Poikonen	Peijaksen Hospital
	Mervi Putkonen	Turku University Hospital
	Anu Räsänen	Kymenlaakso Central Hospital
	Anu Sikiö	Keski-Suomi Central Hospital
	Risto Sippola	Lapland Central Hospital
	Merja Suominen	Kanta-Häme Central Hospital
	Marjaana Säily	Oulu University Hospital
	Venla Terävä	Tampere University Hospital
	Mirja Vapaatalo	Hyvinkää Hospital
	Marko Vesanen	Satakunnan Hospital

SIGNATURE PAGE of the Sponsor, Coordinating Investigator and Principal Investigator's

By my signature, I agree to personally supervise the conduct of this study and to ensure its conduct in compliance with the protocol, informed consent, IRB/EC procedures, the Declaration of Helsinki, ICH Good Clinical Practices guideline, the EU directive Good Clinical Practice, and local regulations governing the conduct of clinical studies.

.... 05 NOV 2012

Signature | Date

Dr Seppo Lehto
Representative of the sponsor

.... 05 NOV 2012

Signature | Date

Dr Raija Silvennoinen
Coordinating Investigator

Principal Investigator Signature

Site name: ....

....

Signature | Date

....

Printed name

SCHEME OF STUDY

REGISTRATION

(randomization for mobilization arm)

3 X RVD

RESPONSE ASSSESSMENT (AT LEAST SD)

MOBILIZATION

$CD34^+$ GOAL $\geq 3 \times 10^6$/kg / for one transplant

ARM A) CY 2 g/m^2 + filgrastim 5 µg/kg subcutaneously on d +4 onwards

ARM B) Filgrastim 10 µg/kg alone starting on d+1 (Thursday) (plerixafor if needed)

RESPONSE ASSESSMENT (AT LEAST SD)

PCR ASSESSMENT IF sCR (normal S-FLC ratio and negative flow cytometry with sensitivity of < 0.01%)

ASCT WITH HDMEL 200mg/m^2 + filgrastim 5 µg/kg if $CD34^+$ cell content < 3×10^6/kg

RESPONSE ASSESSMENT 3 MONTHS POST-ASCT

PCR ASSESSMENT IF sCR

LENALIDOMIDE MAINTENANCE 10mg/d on days 1 – 21,

ONE WEEK OFF, FOR ALL PATIENTS UNTIL PROGRESSION OR TOXICITY

RESPONSE ASSESSMENT EVERY 3 MONTHS DURING THE FIRST YEAR

EVERY 4 MONTHS DURING THE SECOND YEAR

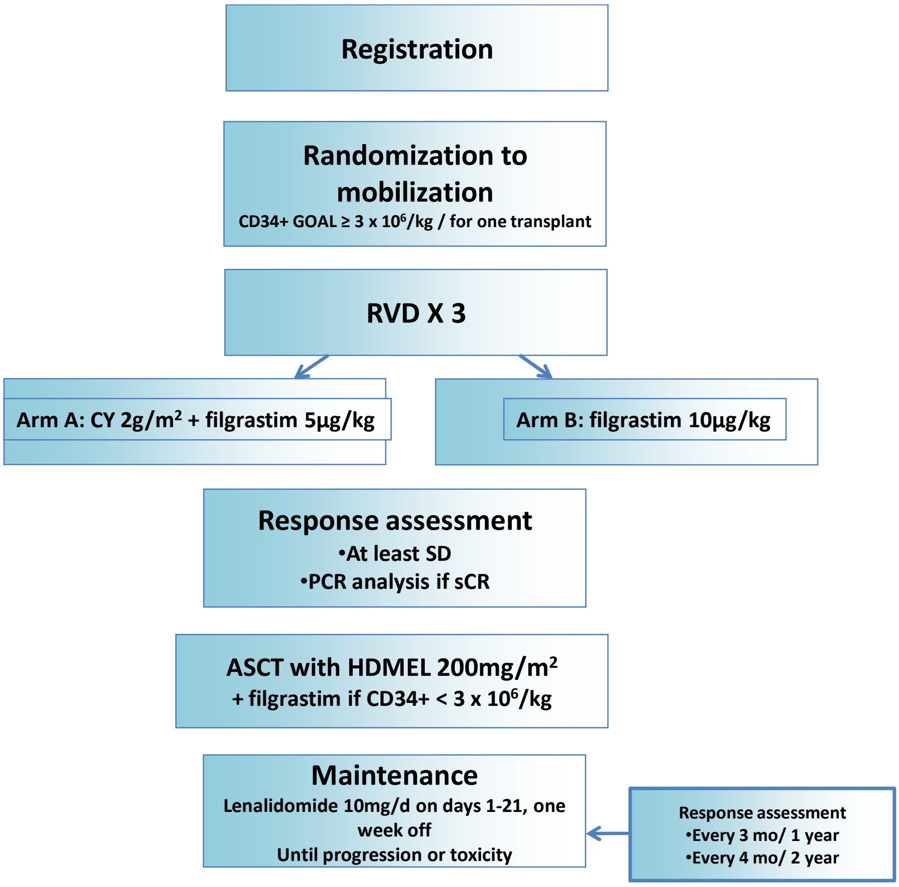

TABLE OF CONTENTS

		PAGE
	Title of study	1
	List of investigators	1
	Signature page	3
	Scheme of study	4
	Table of contents	6
	Synopsis	7
1	Introduction	8
2	Autologous stem cell transplantation	9
3	Immunomodulatory therapy pre and post ASCT setting	9
4	Induction	9
5	Stem cell mobilization	10
	5.1 Randomized study on mobilization	11
	5.2 Graft analysis	14
6	Maintenance	15
7	Rationale of the study	16
8	Study objectives	17
9	Treatment	19
10	Stem cell mobilization and collection	23
11	Maintenance with lenalidomide	24
12	Other medication	25
13	End of protocol treatment	26
14	Clinical evaluations	26
15	Study drug information	26
16	Study investigations, schedule	29
17	Specific additional investigations	31
18	Minimal residual disease	32
19	Toxicity	33
20	Adverse event reporting	37
21	Adverse events updates	43
22	SUSAR	43
23	Annual safety report	43
24	Data safety and monitoring	44
25	Case report forms	44
26	Statistical considerations	44
27	Ethics	46
28	Patient information and consent	46
29	Patient confidentiality	47
30	Study insurance	47
31	Monitoring	47
32	Data and documents handling	47
33	Storage of samples	48
34	Amendments	48
35	Annual reporting and updates	48
36	Publication policy	48
37	Flow cytometry protocol	48
38	Instructions for DNA samples and biobanking	48
39	Study completion and termination	49
40	Financing	49
	Appendix 1 IMWG criteria	50
	Appendix 2 WHO/NYHA	53
	Appendix 3 Neuropathy	54
	References	55

Synopsis

**Study phase**	Phase II
**Primary study objectives**	Complete response rate (including nCR/CR rate, **immunophenotypic remission of nCR/CR patients** and molecular remission of patients in immunophenotypic remission/stringent CR) after induc tion treatment (3 cycles) and 3 months after ASCT<br>Improvement of responses during lenalidomide maintenance (first two years on treatment)<br>Progression-free survival after study inclusion (comparison to VelDex-study)<br>Mobilization study: proportion of patients collected with > 3 x 10^6/kg $CD34^+$ with ≤ 2 aphereses after mobilization with CY 2 g/m^2 + filgrastim (group A) or filgrastim alone (group B)
**Patient population**	Patients with symptomatic multiple myeloma, previously untreated, age 18 – 70 years
**Study design**	Prospective, multicenter, academic, open label, randomized mobilization phase
**Duration of treatment**	Expected duration of treatment is induction 3 months, stem cell collection and ASCT 2 months, lenalidomide maintenance will be started at 3 months after ASCT.<br>Lenalidomide maintenance is planned until progression/ discontinuation due to adverse events.
**Number of patients**	80 patients will be registered
**Expected duration of accrual**	2 years
**Adverse events**	Adverse event will be documented if observed, mentioned during open questioning, or when spontaneously reported
**End of trial**	The study will be closed when the last patient has been two years on maintenance treatment.

1. INTRODUCTION

Multiple myeloma (MM) is the most common hematological malignancy after lymphoma. The annual incidence rate in the Western countries is 4-6/100 000. In Finland every year 250-300 new myeloma patients are registered in the Finnish Cancer Registry giving an incidence of 5.5/100 000/year. Multiple myeloma is an incurable disease. High-dose therapy supported by autologous stem cell transplantation (ASCT) is the standard therapy for multiple myeloma (MM) in eligible patients under 65 years (1). The importance of the traditional complete response of ASCT for OS is confirmed in a meta-analysis (2). Several studies have demonstrated the correlation between low minimal residual disease (MRD) after treatment and improved outcome in MM (3-12). Both of allele specific quantitative polymerase chain reaction, ASO-PCR, and multicolour flow cytometry (MFC) have been used in MM to assess MRD (13-16). ASO-PCR seems to be more sensitive but is quite demanding and time-consuming compared to MFC (13). An individually designed ASO-PCR probe is mandatory for assessing the MRD with ASO-PCR. In previous publications the success rate for designing a suitable probe has been between 75 and 88% (6,11-13). In flow cytometry the aberrant immunophenotype detected at diagnosis is used as a patient-spesific probe for later MFC-MRD analyses.

VAD has been replaced as induction treatment by novel agents; thalidomide- bortezomib- or lenalidomide- based treatments. Several phase 2-3 randomized studies have been performed or are ongoing to compare the responses of different combinations (17-25). Overall response rates up to 94-100% have been reported for lenalidomide plus bortezomib plus dexamethasone (RVD) (26-29). So far only few data has been published regarding MRD after these new induction combinations followed by ASCT (10,12,25) and until now there is no data concerning MRD data after RVD followed by ASCT. The disease will still progress frequently even after CR, and the treatment-free interval will be shorter after each relapse with influence on quality of life. Several randomized studies are ongoing to find out the role of consolidation and maintenance treatment after ASCT (30-34). Two of these have reported similar statistically significant improvements in PFS for lenalidomide maintenance compared to placebo (30-31) No data has been published regarding the MRD measured by FCM or ASO-PCR during lenalidomide maintenance. In the first study of Finnish Myeloma Group we assessed MRD by FCM and ASO-PCR after VD plus ASCT (35). Immunophenotyping is now possible in every university hospital in Finland, and molecular genetics will be centralized in Turku University Hospital. In this first Finnish study ASO-PCR probe was possible to design for every patient in near complete remission/complete remission (nCR/CR) response, which is more than in previous publications (6,11-13). The aim of this new study is to assess complete response rates including immunophenotypic responses of patients in nCR/CR, and molecular responses of patients in confirmed immunophenotypic remission, after RVD induction and after ASCT and during lenalidomide maintenance.

2. AUTOLOGOUS STEM CELL TRANSPLANTATION (ASCT)

Autologous stem cell transplantation as a strategy for myeloma treatment is the standard of care for patients less than 65-70 years of age who can safely undergo the procedure. Successful peripheral blood stem cell (PBSC) mobilization remains a key factor for ASCT. High-dose therapy supported by (ASCT) has been shown to be superior to conventional chemotherapy in patients with newly diagnosed MM (36-42). In two randomized studies both complete remission (CR), event-free survival (EFS) and overall survival (OS) were superior to ASCT (36-37), while in other studies superiority was observed only for CR achievement (38-42). The IFM90 trial was the first to demonstrate the significant impact of CR/VGPR on long-term survival (36).

Regarding the five randomized trials (43-47) comparing single versus tandem double transplants, a significantly longer OS with a double transplant was observed in only one study, but only in patients not in CR or very good partial remission (VGPR) after the first ASCT (43). In two studies there was no survival benefit from tandem transplants (45-46), and in the remaining two only an EFS benefit was observed (44,47). In Finland, seventy-five ASCTs are performed every year for MM of which very few are double ASCTs.

However, ASCT is not curative, and most patients will progress within 2-3 years (1). Double autologous transplants and re-transplants may be performed for extending remission. There is a need for improving the outcomes of ASCT and novel agents such as thalidomide, bortezomib and lenalidomide are now actively investigated both in pre- and post-induction setting.

3. IMMUNOMODULATORY THERAPY PRE AND POST ASCT SETTING

The anti-MM effect of immunomodulatory drugs is thought to be mediated by several biological pathways. Lenalidomide has tumoricidal effect that occurs through several biological mechanisms, including disruption of stromal support, induction of tumor suppressor genes, and activation of caspases. The immunomodulatory effects include T-cell and natural killer (NK)-cell activation, and increased expression of death effector molecules, leading to enhanced immune cell function. The latter may explain partially the beneficial effects of lenalidomide monotherapy in the longer treatments, such as maintenance in newly diagnosed MM patients (48-49)

4. INDUCTION

The combination of lenalidomide, bortezomib, and dexamethasone (RVD) has demonstrated substantial activity in frontline MM patients (26-29). Up to 100% of patients treated at the defined phase 2 dose level responded to treatment: 74% of patients experienced a 90% reduction in tumor burden and 57% entered a nCR/CR response within a few months starting treatment (28). This drug combination is complicated by adverse events, most common neuropathy (sensory neuropathy 80%, painful sensory neuropathy in 32% of patients). Fatigue, constipation, edema

limb, muscle pain, rash, diarrhea and nausea were the next common adverse events. In total, 39%, 42%, and 35% of patients received all the doses or bortezomib, lenalidomide, and dexamethasone, respectively, at the planned dose. In this study, still, 59% of patients received at least 8 cycles of all agents. Dose reduction guidelines are essential for avoiding toxicity but keeping patients on treatment. Thrombosis prophylaxis is also mandatory. In this study incidence of thrombotic adverse events was 6% in spite of aspirin or alternative anticoagulation for prevention of deep-vein thrombosis.

5. STEM CELL MOBILIZATION

Currently more than 99 % of the autotransplants are performed with blood stem cell support. Initially cyclophosphamide (CY) 4-7 g/m^2 was used to mobilize stem cells but subsequently filgrastim and to lesser extent sargramostim were added to enhance mobilization. In retrospective comparison low-dose cyclophosphamide (1.2-2 g/m^2) was comparable in terms of mobilization efficacy but was associated with less toxicity and less need for supportive care (50). Subsequently, CY 2 g/m^2 + G-CSF have become the mobilization standard in the Finnish transplant centres. Especially in USA, but also in some European countries the majority of stem cell mobilizations are currently performed with G-CSF alone. G-CSF mobilization may be cheaper and is less toxic than chemomobilization but is associated with lower yields of CD34$^+$ cells per apheresis (51). In general, chemomobilization with cyclophosphamide is excellent mobilizer in MM patients. In a recent single centre experience only 3 % of patients failed to reach the minimum collection target of 2 x 10^6/kg CD34$^+$ cells (52).

Only one randomized study comparing CY 6 g/m^2 plus G-CSF compared to G-CSF alone including 44 patients is available (53). This study concluded that chemomobilization was more effective but was associated with higher toxicity when compared to G-CSF alone mobilization. There was no difference in engraftment between these groups. The relevance of this randomized trial in regard to current practices is questionable as such high doses of CY are not used any longer for mobilization purposes. Further, the first line therapy in MM patients eligible for ASCT and new drugs may have effect on mobilization capacity (54).

Because cyclophosphamide usually do no improve substantially the treatment response in MM patient after induction therapy (50,55-56), G-CSF mobilization is currently becoming more popular. The most recent mobilization method currently available is the combination of G-CSF and plerixafor, which has been shown in a prospective phase III study to be more effective than G-CSF alone (57). More widespread use of this mobilization method needs, however, studies on cost-effectiveness when compared to other methods currently available. In one retrospective study the costs of G-CSF plus plerixafor were comparable with CY (3-5 g/m^2) plus G-CSF (58). There are no European cost-efficacy analyses in myeloma patients in this regard. While G-CSF plus plerixafor is approved for mobilization of stem cells in MM and lymphoma patients, in Europe plerixafor use is restricted by EMA only to patients who mobilize poorly.

It is reasonable suggest that both CY 2 g/m^2 + filgrastim µ5 mg and filgrastim 10 µg alone are both efficient mobilizers of stem cells although patients mobilized with filgtastim alone may need more apheresis sessions. Pre-emptive use of plerixafor might be an option in those patients who appear to mobilize poorly with either method (59). Some guidelines for pre-emptive use have been suggested (52,59-60). Day 5 plerixafor use in poor mobilizers with filgrastim has been shown to be feasible and efficient (61). It is to be expected that three RVD (lenalidomide, bortezomib, dexamethasone) may have some effect on mobilization capacity. This may imply that a significant proportion of the patients mobilized with filgrastim alone may need plerixafor pre-emptively. Such approach has been shown to be both feasible and efficacious in the context of filgrastim mobilization.

The optimum number of infused $CD34^+$ cells in autologous setting has not been properly determined.. In addition to the number of $CD34^+$ cells given to the patients, also other cellular components like various lymphocyte populations or more primitive stem cells may be important (62). There are no prospective studies in MM patients evaluating graft cellular composition other than $CD34^+$ cell counts in relation to mobilization method used or immunological reconstitution after high-dose therapy.

5.1 RANDOMIZED STUDY ON MOBILIZATION

SUBSTUDY: G-CSF ALONE COMPARED TO LOW-DOSE CYCLOPHOSPHAMIDE PLUS G-CSF FOR MOBILIZATION OF BLOOD STEM CELLS IN MYELOMA PATIENTS: A RANDOMIZED STUDY OF THE FINNISH MYELOMA GROUP

5.1.1 The purposes of the study

The main purpose of this prospective study is to compare the current standard mobilization in Finland (CY 2 g/m^2 + G-CSF) with G-CSF alone (filgrastim in both study arms) in MM patients. In addition to efficacy of mobilization, also engraftment (including lymphoid engraftment) will be evaluated. Also graft content and cost analysis (mobilization and collection phases) will be evaluated.

5.1.2 Endpoints of mobilization study

Primary endpoint:

- percentage of patients reaching ≥ 3 x 10^6/kg $CD34^+$ cells (> 6 x 10^6/kg if two transplants are considered) with ≤ 2 apheresis

Secondary endpoints:

- percentage of the patients reaching ≥ 2 x 10^6/kg CD34$^+$ cells (minimum collection target) with ≤ 3 aphereses
- percentage of the patients receiving plerixafor
- the number of apheresis needed to reach > 3 x 10^6/kg CD34$^+$ cells
- time to platelet (> 20 x 109/L without transfusional support)and neutrophil engraftment (> 0.5 x 10^9/L) after melphalan 200 mg/m^2
- absolute lymphocyte count (ALC), platelet count and neutrophil counts at d+14, and at 1, 3, 6, and 12 months from the stem cell infusion

5.1.3 Additional issues to be analyzed:

- *costs*
    - medication (filgrastim, plerixafor)
    - cost of apheresis including laboratory costs at stem cell laboratory
    - in-hospital days during mobilization and collection
    - no. of platelet transfusions
    - fever associated with mobilization, no. of days with antibiotics
    - number of B-CD34$^+$ determinations/patient
- *graft content*
    - lymphocyte subsets, CD34$^+$ cells after freezing, CD34$^+$ subsets (see later), dendritic cells
    - immunological recovery after stem cell infusion (see later)

5.1.4 Inclusion criteria

- MM needing therapy
- age ≤ 70 years
- informed written consent

5.1.5 Randomization:

- after informed written consent randomization within each study centre (university hospital)

5.1.6 Number of patients

- as 80 patients will be included in the phase II clinical trial of the Finnish Myeloma Groups (VRD induction, ASCT, lenalidomide maintenance), the number of randomized patients will be 40 patients per study arm
- in regard to primary efficacy point it is not likely that a statistically significant difference could be observed even with larger patient numbers
- study size is considered adequate in regard to secondary endpoints

- graft content studies as well as studies regarding immunological reconstitution are costly which also limits the number of patients included

5.1.7 Group A (low-dose cyclophosphamide + filgrastim)

Intravenous fluids 2000 ml before start of cyclophosphamide
Cyclophosphamide 2 g/m^2 in a 90 min infusion on Saturday (d 1)
Uromitexan 800 mg/m^2 3, 6, and 9 h after start with cyclophosphamide
Intravenous fluids 4000 ml with 24 h after cyclophosphamide
5-HT blocking agent plus dexamethasone 20 mg intravenously before cyclophosphamide infusion
Filgrastim 5 µg sc on day 4 onwards
Day 10 (Monday) complete blood count plus B-$CD34^+$ determination at 6 a.m.
Start of apheresis if B-$CD34^+ > 10 \times 10^6$/L

Note: if cyclophosphamide for local logistic reasons cannot be given on Saturday, it may be given on Sunday or Monday, but monitoring of B-$CD34^+$ cell counts should start on day 10 morning (day 1=day of cyclophoshamide infusion)

5.1.8 Group B (filgrastim alone)

Filgrastim 10 µg sc on Thursday at 8-9 a.m. (day 1)
Day 5 (Monday) complete blood count plus B-$CD34^+$ determination at 6 a.m.
Start of apheresis if B-$CD34^+ > 10 \times 10^6$/L

5.1.9 Use of plerixafor

- group A, if B-$CD34^+ < 10 \times 10^6$/L and WBC $\geq 10 \times 10^6$/L and rising after nadir (60)
- group B, if B-$CD34^+ < 10 \times 10^6$/L on Monday morning (d+5)
- in both groups if LA-$CD34^+$ of the first apheresis is $< 1 \times 10^6$/kg
- plerixafor 240 µg/kg sc on 9-10 p.m., start of collection next morning
- if plerixafor is started, continue daily until $\geq 3 \times 10^6$/kg $CD34^+$ cells collected

Complete blood counts at d +14, 1 mo, 3 mo, 6 mo, 12 mo from ASCT

Immunological reconstitution: 1 mo, 3 mo, 6 mo, from ASCT

- flow cytometry CD3/CD8/CD45/CD4 and CD3/CD16 + CD56/CD45/CD19

5.2 GRAFT ANALYSIS

5.2.1 CD34$^+$ subclasses

CD34$^+$ cell number in the graft will be determined as a clinical practice after collection.Other graft studies will be performed from freezed specimen at the Laboratory of Eastern Finland, Kuopio, Finland. The cryopreserved leukapheresis products are thawed in +37 water bath just before flow cytometric analysis. The percentage of CD34$^+$ cells as well as the CD34$^+$ cell content of cryopreserved leukapheresis products are analyzed by flow cytometry (FACSCanto, Becton Dickinson, San Jose CA, USA) using ISHAGE protocol with a single platform method (Stem-Kit, Immunotech SAS/Beckman Coulter, France). 7-aminoactinomycin D (7-AAD) is used to exclude dead cells from the analysis. The same samples from cryopreserved leukapheresis products are also stained for CD34$^+$ subtyping with antibodies (Ab) according to standard procedures and analyzed by FACSCanto flow cytometer. Ab against the following cell-surface markers are used (clone designations in parenthesis): CD34 (8G12), CD38 (HB7), CD133 (AC133), and CD45 (2D1). All antibodies are obtained from Becton Dickinson (Heidelberg, Germany) except for CD133 (Miltenyi Biotec GmbH, Bergisch Gladbach, Germany). The data will be analyzed using BD FACSDIVA software (Becton Dickinson, San Jose, CA, USA).

5.2.2 Lymphocyte subsets

BDMultitest CD3/CD8/CD45/CD4 and CD3/CD16 + CD56/CD45/CD19 reagents (Becton Dickinson, San Jose, CA, USA) with BD Trucount tubes (Becton Dickinson, San Jose, CA, USA) are used wth FACSCanto flow cytometer to determine the absolute counts of T, B, and natural killer (NK) cells as well as CD4 and CD8 subpopulations in the graft (63). The cell content of the graft will be calculated by multiplying the concentration of each cell subpopulations with the graft volume and dividing that by patient weigh (kg). The viability of lymphocytes is measured by using 7-AAD staining.

5.2.3 Dendritic cells

The plasmacytoid dendritic cells are determined by staining the samples from cryopreserved leukapheresis products wth antibodies against the following cell-surface markers (clone designations in parenthesis): CD3 (SK7), CD123 (9F5), CD303 (BDCA-2), HLA-DR (L243) and CD45 (2D1). All antibodies are obtained from Becton Dickinson (Heidelberg, Germany) except CD304 (Miltenyi Biotec GmbH, Bergish Gladbach, Germany). The samples will be analyzed using a FACSCanto flow cytometer using a BD FACSDIVA software. The proportion of plasmacytoid dendritic cells (CD303$^+$, CD123$^+$, HLA DR$^+$) (%) in relation to the CD3+ T lymphocytes in the graft is multiplied with the total CD3+ cell content of the graft (x 10^6/kg) to determine the absolute amount of plasmacytoid dendritic cells in the graft (x 10^6/kg).

5.2.4 GFU-GM assay

In vitro growth of granulocyte/macrophage progenitors (GM-CSF) will be assessed from all grafts. The samples from cryopreserved leukapheresis products will be thawed at + 37 C water bath and suspended in ID-MEM with l-glutamine (Gibco, Paisley, UK). The cell suspension will be dispensed into methysellulose medium containing recombinant human cytokines (MethoCult GF H4434, Stem Cell Technologies, Vancouver, British Columbia, Canada). The methylcellulose medium was aliquoted into two Petri dishes and the dishes are incubated in a humidified 37 C incubator with 5 % CO_2 for a total of 14 days and read using an inverted microscope. CFU-GMs will be enumerated in both dishes, the results are averaged and used to calculate the absolute number of CFU-GM per patients weight (kg).

5.2.5 Lymphocyte subsets in blood after autologous stem cell transplantation

The samples from peripheral blood are taken at 1, 3 and 6 months post-transplant (three time points). Flow cytometry analysis including BD Multitest CD3/CD8/CD45/CD4 and CD3/16 + CD56/CD45/CD19 reagents (Becton Dickinson, San Jose, CA, USA) are used with FACSCanto flow cytometer to determine the absolute proportion of T, B and NK cells as well as CD4 and CD8 subpopulations in the blood samples. These studies will be performed from fresh samples at hematological laboratories of participating university hospitals.

5.2.6 Costs

Analysis of graft cellular content and flow cytometry of blood (x 3) takes about 2000 EUR/patient. Therefore these analyses will be made in only 15-20 patients in both randomized groups (40 patients) which costs about 60000-80000 EUR altogether.

6. MAINTENANCE

New treatment options have been developed for maintaining remissions in patients with MM following ASCT. International Myeloma Working Group (IMWG) consensus on maintenance therapy in multiple myeloma was recently published (64). This report concluded that based on current data available, thalidomide maintenance treatment after ASCT is a possible option that increases PFS and also OS. Bortezomib maintenance seems to improve PFS and OS is patients with adverse FISH-determined cytogenetics, such as deletion of 17p13 and translocation of 4;14 (64,65).

Lenalidomide is associated with a significantly increased PFS in transplant eligible (30,31) and elderly patients (32), and also shows a significant survival benefit (30). The rationale of maintenance therapy after ASCT is that lenalidomide prolongs the time to disease progression. Lenalidomide has moderate side-effect profile. For maintenance, the starting dose of 10 mg was selected based on a high probability of hematologic tolerability following ASCT (Investigator's

Brochure, Celgene Corporation). Patients who did not have cumulative myelosuppression, dose escalation to 15 mg daily was allowed in earlier studies.

7. RATIONALE OF THE STUDY

This is a phase II study to test the efficacy and feasibility of 3-drug combination as induction before ASCT. The efficacy of lenalidomide plus bortezomib plus dexamethasone (RVD) has been shown in earlier studies (26-29). ASCT has been standard treatment in all Western countries in transplant eligible patients under 65- 70 years of age. Two wide international studies are now ongoing comparing intensive versus non-intensive arm: IFM – DFCI 2009 study has RVD as induction, HOVON 95 MM has VCD as induction. Four-drug combination has been tested by Kumar (29), and it seemed that overall response rate could not be improved by adding fourth drug because of increasing toxicity. The depth of complete response has been showed to be important for better outcome, and there are not many studies assessing immunophenotypic remission after RVD induction followed by ASCT. In addition, there is no data concerning the immunophenotypic response during lenalidomide maintenance. RVD is an expensive drug combination and it is important to assess the depth and quality of response before ASCT. The earlier Finnish myeloma study (VD+ASCT) included bortezomib plus dexamethasone induction, so we have historical control group with minimal residual disease data for this FMG-MM-02 study.

Efficacy and toxicity of subcutaneous use of bortezomib has evaluated by IFM (66). This was a study for relapsed patients. Subcutaneous bortezomib was as effective as intravenous with less side effects, especially grade 3-4 polyneuropathy. In this study subcutaneous bortezomib will be tested as part of induction treatment, which is a novel approach.

If mobilization of autologous stem cells is performed after 3-4 induction cycles, it is usually successful according to IMWG (54,67), if lenalidomide is used more than 4 cycles, mobilization with cyclophosphamide (CY) is recommended. Timing of collection days would be better predicted with granulocyte growth factor mobilization. Avoiding of extra alkylating agents during mobilisation may be important for myeloma patients. Inside this study we have a mobilization substudy with randomization to CY 2g/m2 + filgrastim 5µg/kg or filgrastim 10 µg/kg alone (plerixafor if needed).

Two studies have shown that lenalidomide maintenance can improve progression-free survival significantly after ASCT and in the study of McCarthy et al. also the OS benefit is noticed (30-31). However, the question about risk of second primary malignancies (SPM) has arisen. It seems that there is some association with the load of alkylating agents the patient has received, and with age, male sex and ISS of patient as well. In this study we have different dosing strategy for lenalidomide maintenance than in earlier studies (30,31), namely intermittent dosing with three weeks on lenalidomide, one week off. During this study we will carefully follow the data

concerning ongoing lenalidomide maintenance studies and the risk of secondary malignancies: amendment of study will be done if ethically necessary.

8. STUDY OBJECTIVES

The primary objective of this study is to determinate the rate of immunophenotypic remission after induction treatment with RVD prior to high-dose melphalan and autologous stem cell transplantation (HDT-ASCT), after HDT-ASCT, and during lenalidomide maintenance in patients with multiple myeloma.

8.1 ENDPOINTS

8.1.1 Primary endpoints:

1) Complete response rate (including nCR/CR rate, **immunophenotypic remission of nCR/CR patients** and molecular remission of patients in immunophenotypic remission/stringent CR) after induction treatment (3 cycles) and 3-4 months after ASCT
2) Improvement of responses during lenalidomide maintenance
3) Progression-free survival after study inclusion (comparison to VelDex-study)

8.1.2 Secondary endpoints:

1) Feasibility of subcutaneous use of bortezomib as a part of induction therapy in myeloma patients
2) Feasibility of three-drug combination induction therapy (discontinuation of therapy, decrease of doses)
3) nCR/CR rate after 2 cycles, at mobilization; 3 months, 6 months, 9, 12, 16, 20, 24 months after ASCT
4) Overall response rate
5) Number of $CD34^+$ cells collected after low-dose CY + G-CSF *vs.* G-CSF alone-mobilization, number of aphereses and costs according to the mobilization arm, graft composition
6) Duration of treatment
7) Overall survival from the study inclusion

8.2 STUDY POPULATION

8.2.1 Inclusion criteria

- Age 18 - 70 years
- Written informed consent
- Patients with a symptomatic, previously untreated, ISS 1-3 multiple myeloma
- Measurable disease: serum paraprotein > 10g/l or urine paraprotein > 200mg/24 hours or abnormal free light chain ratio

- WHO performance status 0-3 (if WHO 3, must be related to myeloma, not due to comorbidities)
- Eligible for ASCT
- Sufficient renal function, glomerular filtration rate (GFR) assessed by Modification of Diet in Renal Disease formula (MDRD) > 15 ml/min unless on hemodialysis
- Women of childbearing potential (WCBP) must have a negative serum or urine pregnancy test prior to starting lenalidomide. In addition, sexually active WCBP must agree to use adequate contraceptive methods (oral, injectable, patches, or implantable hormonal contraceptive methods; tubal ligation; intra-uterine device; barrier contraceptive with spermicide; or vasecomized partner) while on lenalidomide. WCBP must agree to have pregnancy tests every 4 weeks while on lenalidomide.
- Males (including those who have had a vasectomy) must use barrier contraception (latex condoms) when engaging in reproductive sexual activity with WCBP while on lenalidomide, when temporarily stopping lenalidomide and 28 days after the last dose of lenalidomide.

8.2.2 Exclusion criteria

- Previous chemotherapy or radiotherapy except local radiotherapy (10-20 Gy) or corticosteroids maximum dexamethasone 160 mg in two weeks before study inclusion for symptom control
- Peripheral neuropathy grade ≥ 2
- Contraindication to use of thromboembolic prophylaxis
- Significant hepatic dysfunction (serum bilirubin 3 x >ULN, transaminases ≥ 2,5 x upper limit), unless related to myeloma
- Severe cardiac dysfunction
- Severe renal failure, GFR < 15ml/min, if not in hemodialysis
- Other serious medical or psychiatric illness
- Uncontrolled infection
- Patients with a history of active malignancy during the past 5 years with the exception of basal carcinoma of the skin or stage 0 cervical carcinoma
- Pregnancy
- Lactating women
- HIV-positive patients, HBsAg or HCV positive patients
- Primary plasma cell leukemia
- Systemic AL amyloidosis or myeloma associated amyloidosis if not eligible for ASCT
- Allogeneic stem cell transplantation planned
- Participants receiving any other investigational agents
- Participants with known brain metastases

Sperm should be frozen from men, < 40 years of age, with child wish before start of treatment.

9. TREATMENT

The patient should be registrated immediately after diagnosis of symptomatic myeloma, before the start of any study related procedure. Patients will be registered with the Finnish Hematology Register. Each patient will have a unique patient identification number when registered.

The following information will be requested at registration:

- Name of study center
- Name of responsible investigator (treating physician)
- Date of birth of the study patient
- Date of signed informed consent
- Date of diagnosis
- Eligibility criteria met
- Treatment start date

9.1 RVD induction phase

All patients will receive 3 cycles of RVD with subcutaneous bortezomib according this schedule:

Agent	Dose/day	Route	Days
Bortezomib	1.3mg/m^2	Subcutaneous	1, 4, 8, 11
Lenalidomide	25mg	p.o	1 – 14
Dexamethasone	20mg	p.o	1-2, 4-5, 8-9, 11-12

Cycle 2 and 3 will start on 22nd day after preceding cycle.

9.1.1 Dose modifications

Before each cycle, the participant will be evaluated for possible toxicities that may have occurred after the previous dose(s). Toxicities are to be assessed according to the NCI Common Terminology Criteria for Adverse Events (CTCAE Version 4.0). Dose modifications or delays will be done based on the toxicity experienced during a cycle of therapy or newly encountered on day 1 of each cycle.

The participant may continue on therapy if the toxicity can be managed according to the dose modification guidelines as outlined below. Once one of the treatment dose (lenalidomide, bortezomib or dexamethasone) is reduced for toxicity, no re-escalation will be allowed.

9.1.2 BORTEZOMIB – DOSE REDUCTION STEPS FOR BORTEZOMIB

Starting dose of bortezomib	1st dose reduction	2nd dose reduction	3rd dose reduction
1.3 mg/m^2 on days 1, 4, 8, 11	1.0 mg/m^2 on days 1, 4, 8, 11	0.7 mg/m^2 on days 1, 4, 8, 11	Discontinue bortezomib

If there were dose modifications or delays in the previous cycle, use the following guidelines:

- In case of dose reduction during initial RVD therapy, the participant will receive the reduced dose levels (the last level applied during initial therapy).
- If any two or more doses of bortezomib were held during the cycle (either consecutively or two or more in one cycle), then the new cycle will be started with one level dose reduction.
- If the new cycle is delayed due to bortezomib-related toxicity newly encountered on the scheduled Day 1, then the new cycle will be started with a one-level dose reduction.

Recommended*dose modifications for bortezomib related neuropathy.

**Severity of neuropathy**	**Modification of dose and regimen**
Grade 1 (paraesthesia, weakness and/or loss of reflexes) with no pain or loss of function	No action
Grade 1 with pain or Grade 2 (interfering with function but not with activities of daily living)	Reduce to 1.0 mg/m^2
Grade 2 with pain or Grade 3 (interfering with activities of daily living)	Withhold VELCADE treatment until symptoms of toxicity have resolved. When toxicity resolves re-initiate VELCADE treatment and reduce dose to 0.7 mg/m^2 and change treatment schedule to once per week.
Grade 4 (sensory neuropathy which is disabling or motor neuropathy that is life threatening or leads to paralysis)<br>And/or severe autonomic neuropathy	Discontinue VELCADE

*Based on dose modifications in phase II & III multiple myeloma studies and post-marketing experience.

Use of bortezomib in multiple myeloma patients with renal impairment
The safety profile is not affected in patients with renal impairment versus those with normal renal function. Since dialysis may reduce bortezomib concentrations, it should be administered after the dialysis procedure.

Dosage in Patients with Hepatic Impairment
Patients with mild hepatic impairment do not require a starting dose adjustment and should be treated per the recommended bortezomib dose. Patients with moderate or severe hepatic impairment should be started on bortezomib at a reduced dose of 0.7mg/m^2 per injection during the first cycle, and a subsequent dose escalation to 1.0mg/m^2 or further dose reduction to 0.5mg/m^2 may be considered based on patient tolerance.

Recommended starting dose modification for bortezomib in patients with hepatic impairment

	Bilirubin Level	SGOT (AST) Levels	Modification of Starting Dose
Mild	Less than or equal to 1.0x ULN	More than ULN	None
	More than 1.0x–1.5x ULN	Any	None
Moderate	More than 1.5x–3x ULN	Any	Reduce VELCADE to 0.7 mg/m^2 in the first cycle. Consider dose escalation to 1.0 mg/m^2 or further dose reduction to 0.5 mg/m^2 in subsequent cycles based on patient tolerability.
Severe	More than 3x ULN	Any	

Abbreviations: SGOT = serum glutamic oxaloacetic transaminase;
AST = aspartate aminotransferase; ULN = upper limit of the normal range.

9.1.3 LENALIDOMIDE- DOSE REDUCTION STEPS FOR LENALIDOMIDE

Starting dose adjustment for renal impairment:
Since lenalidomide is primarily excreted unchanged by the kidney, adjustments to the starting dose of lenalidomide are recommended to provide appropriate drug exposure in patients with moderate or severe renal impairment and in patients on dialysis. Based on a pharmacokinetic study in patients with renal impairment due to nonmalignant conditions, lenalidomide starting dose adjustment is recommended for patients with CreaCl < 60 mL/min. Non-dialysis patients with CreaCl less than 11 mL/min, and dialysis patients with creatinine clearances less than 7 mL/min, have not been studied. The recommendations for initial starting doses for patients with multiple myeloma (MM) are as follows:

Starting Dose Adjustment for Renal Impairment		
**Category**	**Renal Function (Cockcroft-Gault)**	**Dose**
**Mild renal impairment**	CLcr ≥50 ml/min	25 mg once daily
**Moderate Renal Impairment**	CLcr 30-50 mL/min	10 mg once daily
**Severe Renal Impairment**	CLcr <30 mL/min (not requiring dialysis)	15 mg every other day
**End Stage Renal Disease**	CLcr <30 mL/min (requiring dialysis)	5 mg once daily.<br>On dialysis days, administer the dose after dialysis

**Starting dose of lenalidomide**	**1st dose reduction**	**2nd dose reduction**	**3rd dose reduction**	**4th dose reduction**	**5th dose reduction**
25 mg x 1 po On days 1-14 every 21 days	20 mg x 1 po on days 1-14 every 21 days	15 mg x 1 po on days 1-14 every 21 days	10 mg x 1 po on days 1-14 every 21 days	5 mg x 1 po on days 1-14 every 21 days	Discontinue lenalidomide

If there were dose modifications or delays in the previous cycle, use the following guidelines:

- If the cycle was completed without requiring further dose modification, then the next cycle will start at the same reduced dose of lenalidomide.
- If lenalidomide was held during the previous cycle and restarted at a reduced dose level, without interruption for the remainder of the cycle, then the reduced dose level will be initiated on Day 1 of the new cycle.
- If lenalidomide dosing was omitted for the rest of the previous cycle or if a new cycle is delayed due to lenalidomide-related toxicity newly encountered on the scheduled Day 1, then the new cycle will be started with one-level dose reduction.

There are currently no recommendations for dose adjustment of lenalidomide in patients with hepatic insufficiency.

9.1.4 Dexamethasone- Dose reduction steps for dexamethasone:

Starting dose of dexamethasone	1st dose reduction	2nd dose reduction
20 mg/d on days 1, 2, 4, 5, 8, 9, 11 and 12	10 mg/d on days 1, 2, 4, 5, 8, 9, 11 and 12	Discontinue dexamethasone

In case of dose reduction during initial RVD therapy, the participant will receive the reduced dose levels (the last level applied during initial therapy).

Laboratory tests before each cycle are described in schedule of investigations.
Other medication: prophylactic treatment for pneumocystis jirovecii and herpes zoster are strongly recommended
The response of patients will be evaluated after 2 and 3 cycles. Patients with progressive disease will be taken off protocol treatment. All other patients will proceed to randomized mobilization phase:

10. STEM CELL MOBILIZATION AND COLLECTION

10.1 Stem cell mobilization

Manufacturers recommend a dose reduction of 25% if the GFR is 10–50 ml/min, and of 50% if GFR is <10ml/min.

Arm A

Agent	Dose/day	Route	Starting day
Cyclophosphamide	2g/m^2	iv (2g/30 min infusion rate)	1
Uromitexan	800mg/m^2, 4 doses (0,3,6,9h)	iv	1
Filgrastim	5 µg/kg	sc	4
Plerixafor if needed			

Arm B

Agent	Dose/day	Route	Starting day
Filgrastim	10 µg/kg	sc	1, Thursday at 8-9 am
Plerixafor if needed			

10.2 Stem cell collection:

The procedure will be performed according to the local standard protocols. If insufficient number of stem cells is collected a new mobilization attempt will be considered with G-CSF plus plerixa-for.

10.3 High dose melphalan and stem cell transplantation

High dose melphalan and stem cell transplantation will be scheduled in 3-4 weeks after stem cell collection.

**Agent**	**Dose/day**	**Route**	**Days**
Melphalan	200mg/m2	iv	-2
Stem cell infusion	At least 2 x 10e6/kg	iv	0
Filgrastim	5 µg/kg	sc	d5 until recovery if CD34+ < 3 x10^6/kg

In case of renal failure the dose of melphalan will be reduced to 140 mg/m^2. The manufacturer recommends that initial doses of melphalan should be reduced by 50% if the GFR is 40-50ml/min.

If the patient has dialysis-dependent renal failure the dose of melphalan is 140mg/m^2 and will be divided given on days -3 and -2.
Stem cell transplantation procedure will be performed according to the local standard protocols.

Tandem transplant is not included in the protocol. Centers which have policy to collect two autologous grafts will store second graft for progression.

11. MAINTENANCE TREATMENT WITH LENALIDOMIDE

Lenalidomide maintenance will be started after assessment of best response to ASCT in 2-3 months after ASCT.

Agent	Dose/day	Route	Days
Lenalidomide	10mg	p.o	1-21

Lenalidomide will start if neutrophil count is ≥ 1.0 x 10^9/l and platelet > 75 x 10^9/l.

11.1 Dose levels for lenalidomide during maintenance therapy

- Starting dose is 10 mg once daily on days 1-21 every 28 days
- Dose level -1 is 5 mg once daily on days 1-21 every 28 days
- Dose level -2 is no lenalidomide

11.2 Dose modification instructions for lenalidomide for haematologic toxicity* during maintenance

Neutropenia
Neutrophil < 0.5 x 10^9/L =grade 4 neutropenia OR febrile neutropenia (fever >/=38.5 °C and neutrophil < 1 x 10^9/L): Stop the dose for remainder of cycle.

If ANC is recovered / febrile neutropenia is resolved start next cycle.
Decrease by 1 dose level when dosing restarts at next cycle.

Thrombocytopenia
Grade 4: Platelets < 25 x 10^9/L
Stop the dose for remainder of cycle. If platelets are recovered start next cycle. Decrease by one dose level when dosing restarts at next cycle.
* Exclude other causes, especially progressive disease.

11.3 Dose modification instructions for lenalidomide for non-haematologic toxicity during maintenance

Rash = Grade 3: Hold dose for remainder of cycle. Decrease by one dose level when dosing restarted at next cycle (rash must resolve to ≤ Grade 1).
Rash = Grade 4 or blistering: Discontinue lenalidomide and discontinue subject from study
Constipation ≥ Grade 3: Hold dose for remainder of cycle. Initiate bowel regimen.
Decrease by one dose level when dosing restarted at next cycle
(Constipation must resolve to ≤ Grade 2).
Thrombosis/embolism ≥ Grade 3: Hold dose for remainder of cycle. Initiate anticoagulation treatment. Maintain dose level when dosing restarted at next cycle at discretion of treating physician.
Hypo/hyperthyroidism ≥ Grade 2: Hold dose for remainder of cycle. Initiate appropriate medical therapy. Maintain dose level when dosing restarted at next cycle at discretion of treating physician
Other non-hematological toxicity: Hold lenalidomide until toxicity resolves to ≤ grade 2 and contact study coordinator. After consultation with the study PI drug may be resumed at lower dose level, as described above.
Other non-hematological toxicity grade 4: Discontinue lenalidomide and contact study PI.

12. OTHER MEDICATIONS

Thrombosis prophylaxis will start according to international guidelines (68-69).
Prophylaxis for PCC and herpes zoster are recommended.

Vaccination after ASCT; a substudy regarding vaccination will be considered

Bisphosphonates will be started at diagnosis of symptomatic myeloma. Intravenous administration of zoledronate 4 mg or pamidronate 30-90 mg once every 4-6 weeks is recommended for maximum of 2 years especially if sCR is achieved.

Erythropoietic agents, or other agents that may increase the risk of thrombosis, such as hormone replacement therapy, should be used with caution in multiple myeloma patients receiving lenalidomide with dexamethasone. A haemoglobin concentration above 120g/l should lead to discontinuation of erythropoietic agents.

Red cell transfusion
Platelet transfusion

- are allowed for clinical reasons
- for thrombocytopenia see the guidelines for hematologic toxicity

13. END OF PROTOCOL TREATMENT

- Normal completion
- Excessive toxicity
- Death
- Progression / relapse
- Patient's own wish to terminate study treatment
- If the responsible physician thinks a change of therapy would be best for the patient
- No compliance of the patient
- Pregnancy

14. CLINICAL EVALUATIONS

Response will be evaluated according to the IMWG criteria (Appendix 1). Disease progression is not to be reported as an adverse event. Progression is defined as one of the efficacy endpoints.

Time of response evaluations

- At entry: before start of induction (skeleton x-ray and cytogenetic tests in 3 months before start and laboratory and bone marrow in 2 weeks before start)
- After 2 RVD
- After 3 RVD
- After mobilization/At ASCT
- After ASCT: 2-3 months after ASCT
- During maintenance first year: with 3 months interval
- Second year: with 4 months interval

15. STUDY DRUG INFORMATION

Description of study drugs

15.1 DRUG INFO LENALIDOMIDE

Lenalidomide is a thalidomide analogue, an immunomodulatory agent with anti-angiogenic properties. The chemical name is 3-(4-amino-1-oxo 1,3-dihydro - 2H-isoindol-2-yl) piperidine-2,6-dione and it has the following chemical structure:

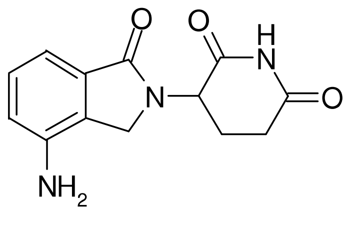

Lenalidomide is off-white to pale-yellow solid powder. It is soluble in organic solvent/water mixtures, and buffered aqueous solvents. Lenalidomide is more soluble in organic solvents and low pH solutions. Solubility was significantly lower in less acidic buffers, ranging from about 0.4 to 0.5 mg/ml. is available in 2.5 mg and 5 mg capsules for oral administration. In this study 25mg, 10mg and 5 mg capsules will be used. The mechanism of action of lenalidomide remains to be fully characterized. Lenalidomide possesses anti-neoplastic, immunomodulatory and anti-angiogenic properties. In vitro, it inhibits secretion of the pro-inflammatory cytokines TNF-α, IL-1β, and IL-6 and increases secretion of the anti-inflammatory cytokine IL-10. It also induces T-cell proliferation and IL-2 and IFN-γ production in vitro. Lenalidomide, in healthy volunteers, is rapidly absorbed following oral administration with maximum plasma concentrations occurring between 0.625 and 1.5 hours post-dose. In multiple myeloma patients maximum plasma concentrations occurred between 0.5 and 4.0 hours post-dose both on Days 1 and 28. The metabolic profile of lenalidomide in humans has not been studied. In healthy volunteers, approximately two-thirds of lenalidomide is eliminated unchanged through urinary excretion. The process exceeds the glomerular filtration rate and therefore is partially or entirely active. Half-life of elimination is approximately 3 hours (Investigator's Brochur, Version 15, Celgene Corporation).

Supplier

Celgene Corporation will supply lenalidomide 5 mg, 10 mg and 25 mg capsules free of charge for the duration of this trial. The drug will be sent to a central pharmacy in Finland. The central pharmacy will be responsible for distributing the drug to the sub-sites. No distribution will take place before required documentation is in place.

Dosage form

Lenalidomide will be administered as 5, 10 or 25 mg capsules to be taken orally.

Packaging

Celgene Corporation will supply lenalidomide 5 mg, 10 mg and 25 mg. Capsules in blisters provided in wallets labelled for clinical trial use. Study drug must be dispensed in the original packaging with the label clearly visible.

Study Medication Recipient and Storage

The investigator(s) or designee(s) is responsible for taking an inventory of each shipment of lenalidomide received, and comparing it with the accompanying lenalidomide accountability form. The investigator(s) will verify the accuracy of the information on the form, sign and date it, retain a copy in the study file, and return to Celgene.

At the study site, all investigational study medications will be stored in a locked, safe area to prevent unauthorized access.

Lenalidomide should be stored at room temperature away from direct sunlight and protected from excessive heat and cold.

Record of Administration

Accurate recording of all study medication administration (including dispensing and dosing) will be made in the appropriate section of the subject's CRF and source documents.

Study Medication Accountability

The investigator(s) or designee(s) is responsible for accounting for all study medication that is issued to and returned by the subject during the course of the study.

Study Medication Handling and Disposal

Investigator or designee will return unused study drugs to pharmacy for destruction. If any study medication is lost or damaged, its disposition should be documented in the subject's CRF and source documents

Storage

At the study site, all investigational study drugs will be stored in a locked, safe area to prevent unauthorized access. The study drug should be stored at room temperature (excursions permitted to + 15- +30°C).

15.2 VELCADE 3.5 mg powder for solution for injection

Each vial contains 3.5 mg bortezomib (as a mannitol boronic ester).

For subcutaneous reconstitution, add 1.4 mL of sterile 0.9% sodium chloride solution to the powder contained in the vial of VELCADE

— This reconstitution will result in a final concentration of 2.5 mg/mL VELCADE

— The reconstituted product should be a clear and colorless solution free of particulate matter

15.3 Dexamethasone will be administered per oral; tablets á 1,5 mg (13 tablets/day) or 4 mg (5 tablets /day).

16. STUDY INVESTIGATIONS
16.1 SCHEDULE OF INVESTIGATIONS

	At entry	At each VRD	On day +11	After 3rd VRD/ At Mob	After Mob/At ASCT	3 mo after ASCT	During maintenance every 3 mo/ 1. yr	During maintenance every 4 mo/ 2. yr	At progr/ relapse
Medical history	X	X		X	X	X	X	X	X
Physical examin.	X	X		X	X	X	X	X	X
Hematology	X	X	X	X	X	X	X	X	X
Immunochemistry	X	X		X	X	X	X	X	X
Blood chemistry	X	X		X	X	X	X	X	X
Blood immunology	X				X	X	**When best response is achieved and lost**		X
Graftanalysis sample				X					
PB FCM**									
Crea clearance	X				X				
BMasp+immuno-phenotype/MRD	X			X		X	X	X	X
Bm biopsy*									
DNA for ASO-PCR (EDTA+CPT tube)	X			X	X	X	X	X	
Skeletal survey	X					X		X	
Neurologic evaluat.	X	X		X	X	X			
ECG	X			X	X				
Thorax-x-ray	X				X				
Sperm cryopreserv.	If need								
PB cryopreserv.	X								
BM cryopreserv.	X								

Pregnancy test [h] at entry and every month thereafter

[h] Women of child-bearing potential (WCBP) only; A woman of childbearing potential is a sexually mature woman who: 1) has not undergone a hysterectomy or bilateral oophorectomy or 2) has not been naturally postmenopausal for at least 24 consecutive months (i.e., has had menses at any time in the preceding 24 consecutive months). Serum or urine beta-human chorionic gonadotrophin (b-HCG) levels will be analysed.

* if inadequate aspiration, biopsy sample for immunohistochemistry and ASO-PCR ** flow cytometry of peripheral blood; 1, 3 and 6 months after ASCT

16.2 Medical history

- complete medical history before induction
- symptoms of illness
- history of thrombosis of the patient and the relatives (before induction)
- performance status (WHO)
- infections
- bone symptoms
- bleeding
- polyneuropathy
- gastrointestinal symptoms

16.3 Physical examination

- Standard physical examination with cardiovascular and neurological (polyneuropathy, autonomic neuropathy) examination
- Orthostatic hypotension
- Body weight and height, surface area
- Exclusion of infections and bleedings

16.4 Hematology

- Hemoglobin, leukocytes, differential count (at dg), neutrophils, platelets

16.5 Blood chemistry

At entry all test, after that creatinine, CRP, alat, bilirubin, alkaline phosphatase, potassium, sodium, ion-calcium are needed (S-HCG if female patient < 50 yr)

- Creatinine
- Liver enzymes (alat, afos, bil)
- Albumin
- Serum B2-microglobulin
- LDH
- CRP
- Ionized calcium, phosphate, sodium, potassium
- Uric acid
- Creatinine clearance

16.6 Biological objectives

Blood, bone marrow and urine samples will be required at diagnosis and at key time-points in follow-up for biochemical, cytogenetic, molecular genetic and immunophenotypic assessments, as part of scientific studies in order to monitor MRD as defined by paraprotein, serum free light chain and flow cytometry to define depth and quality of responses. In addition, cytogenetic FISH findings are utilized as prognostic markers. Immunological studies will performed as separate sub-projects (such as cereblon expression, T-regs measurement etc.).

Peripheral blood flow cytometry 1, 3 and 6 months after ASCT.

Graft analysis (5.2): four samples á 0.5 ml frozen from each graft

16.7 Immunochemistry

Serum- and 24h urine- protein electrophoresis and immunofixation

- For qualification of M-protein at entry and to confirm CR
- Quantification of serum- or urine M-component always when response is assessed
- Serum free light chain assay always when response is assessed

16.8 Bone marrow assessment

Bone marrow aspiration at entry and if nCR/serological CR
Morphology and immunophenotyping
FISH analysis IgH; if positive, screening of translocation of 4;14, 14;16, 14;20, 11;14

- 17 deletion/monosomy, p53 mutation
- 13 deletion/monosomy
- 1q25 gain, 1p36 loss
- amplification chrom 9, chromosome 6 aberration

G-banding (hypodiploidy, hyperdiploidy, 13q del/monosomy)

DNA purification and cryopreservation at entry for ASO-PCR probe designing and assessment of molecular response if sCR/immunophenotypic remission

- Bone marrow sample 3-5 ml- EDTA tube; DNA purification from all nucleated cells
- Bone marrow sample 3-5 ml- CPT tube; DNA purification from mononuclear cells

Bone marrow aspiration if nCR/serological CR

- Morphology and immunophenotyping
- DNA purification and cryopreservation (only EDTA- bone marrow sample)

17. SPECIFIC ADDITIONAL INVESTIGATIONS

- Skeletal survey: skull, pelvis, spina/vertebral column, long bones

- at entry and 3 months after ASCT of affected bones
- during maintenance and follow-up skeletal survey will be performed after 12-18 months and whenever clinically indicated

- Cardiological investigations, echo, if clinically needed
- Spirometry and diffusion capacity, if clinically needed
- MRI, PETCT (not routinely, performed for clinical reasons and considered if extrame-dullary disease)

18. MINIMAL RESIDUAL DISEASE

Several trials have demonstrated the prognostic impact of MRD levels in multiple myeloma after and during therapy. MRD levels were shown to be predictive for PFS and OS irrespective of IF status (10-12). MRD status in multiple myeloma has been analyzed in heavily treated patients, usually with HDMEL and ASCT. The study by Paiva et al. demonstrated that MRD was the strongest predictor for PFS and OS in multivariate analysis using 4-color flow cytometry (14). MRD measured both prior to and after autologous stem cell transplantation were predictive for OS and PFS. The demonstration of the prognostic impact of MRD have led to the novel response category "immunophenotypic CR" and molecular remission by the International Myeloma Workshop Panel (70).

18.1 Immunophenotyping

At diagnosis, at mobilization, 2-3 months after ASCT and during the maintenance treatment a bone marrow aspirate will be taken for both morphology and immunophenotyping. Immunophenotyping is mandatory for patients in immunofixation negative CR with normal free light chain ratio to confirm stringent CR (sCR, no clonal plasma cells).

For multiparameter flow cytometry bone marrow samples are stained with relevant antibodies, and analyzed with FacsCanto, FacsCantoII or Navios flow cytometers for six, eight and ten colour protocols, respectively. At diagnosis, the mandatory antibodies are CD138, CD38, CD45, CD56, CD19, CD81, CD200, CD27, CD28, CD117, CD20 and intracytoplasmic kappa and lambda and the optional antibodies FGFR3 and CD229. The number of events acquired should be at least 1000 000. In follow-up samples (to confirm stringent CR, measure the amount of residual clonal plasma cells and the proportion on normal polyclonal plasma cells or detect immunophenotypic remission) relevant combinations of the informative antibodies are used. The aim should be to acquire at least 1 000 000 events (minimum sensitivity of 50 / 1 000 000 = 0.005%).

18.2 Molecular investigations:

Bone marrow sample will be stored at diagnosis, after induction phase and 3 months after ASCT. If the stringent CR/ immunophenotypic remission has been reached allele-specific oligonucleotide- probe will be designed and also the MRD-PCR assessment will be started by the protocol.

19. TOXICITY ASSESSMENT

Most common side effects of treatment are cytopenia, infection and bleeding.

19.1 BORTEZOMIB

The most commonly reported adverse reactions during treatment with VELCADE are nausea, diarrhoea, constipation, vomiting, fatigue, pyrexia, thrombocytopenia, anaemia, neutropenia, peripheral neuropathy (including sensory), headache, paraesthesia, decreased appetite, dyspnoea, rash, herpes zoster and myalgia. Serious adverse reactions uncommonly reported during treatment with VELCADE include cardiac failure, tumour lysis syndrome, pulmonary hypertension, reversible posterior leukoencephalopathy syndrome (RPLS), acute diffuse infiltrative pulmonary disorders and rarely autonomic neuropathy.

Gastrointestinal toxicity: Gastrointestinal toxicity, including nausea, diarrhoea, vomiting and constipation are very common with VELCADE treatment. Patients who experience constipation should be closely monitored.

Haematological toxicity: VELCADE treatment is very commonly associated with haematological toxicities (thrombocytopenia, neutropenia and anaemia). The most common haematologic toxicity is transient thrombocytopenia. Platelets were lowest at Day 11 of each cycle of VELCADE treatment. VELCADE therapy should be withheld when the platelet count is <25,000/µl and re-initiated at a reduced dose after resolution. Complete blood counts (CBC) including platelet counts should be frequently monitored throughout treatment with VELCADE.

Peripheral neuropathy: Treatment with VELCADE is very commonly associated with peripheral neuropathy, which is predominantly sensory. However, cases of severe motor neuropathy with or without sensory peripheral neuropathy have been reported. The incidence of peripheral neuropathy increases early in the treatment and has been observed to peak during cycle 5.

It is recommended that patients be carefully monitored for symptoms of neuropathy such as a burning sensation, hyperesthesia, hypoesthesia, paraesthesia, discomfort, neuropathic pain or weakness. Patients experiencing new or worsening peripheral neuropathy should undergo neurological evaluation and may require the dose and schedule of VELCADE to be modified (see section 9.2.1 and Appendix 3). In addition to peripheral neuropathy, there may be a contribution of autonomic neuropathy to some adverse reactions such as postural hypotension and severe constipation with ileus.

Hypotension: VELCADE treatment is commonly associated with orthostatic/postural hypotension. Most undesirable effects are mild to moderate in nature and are observed throughout treatment. Caution is advised when treating patients with a history of syncope receiving medicinal

products known to be associated with hypotension; or who are dehydrated due to recurrent diarrhoea or vomiting. Patients should be instructed to seek medical advice if they experience symptoms of dizziness, light-headedness or fainting spells.

Reversible Posterior Leukoencephalopathy Syndrome (RPLS): There have been reports of RPLS in patients receiving VELCADE. RPLS is a rare, reversible, rapidly evolving neurological condition which can present with seizure, hypertension, headache, lethargy, confusion, blindness, and other visual and neurological disturbances.

Heart failure: Acute development or exacerbation of congestive heart failure, and/or new onset of decreased left ventricular ejection fraction has been reported during bortezomib treatment. Fluid retention may be a predisposing factor for signs and symptoms of heart failure.

ECG investigations: There have been isolated cases of QT-interval prolongation in clinical studies, causality has not been established.

Pulmonary disorders: There have been rare reports of acute diffuse infiltrative pulmonary disease of unknown aetiology such as pneumonitis, interstitial pneumonia, lung infiltration, and acute respiratory distress syndrome (ARDS) in patients receiving VELCADE. Some of these events have been fatal. A pre-treatment chest radiograph is recommended to determine if any additional diagnostic measures are necessary and to serve as a baseline for potential post-treatment pulmonary changes. In the event of new or worsening pulmonary symptoms (e.g. cough, dyspnoea), a prompt diagnostic evaluation should be performed.

Renal impairment: Renal complications are frequent in patients with multiple myeloma. Patients with renal impairment should be monitored closely.

Hepatic impairment: Bortezomib is metabolized by liver enzymes. Bortezomib exposure is increased in patients with moderate or severe hepatic impairment; these patients should be treated with VELCADE at reduced doses and closely monitored for toxicities (see section 9.2.1).

Tumour lysis syndrome: The patients at risk of tumour lysis syndrome are those with high tumour burden prior to treatment. These patients should be monitored closely and appropriate precautions taken.

Concomitant medicinal products: Patients should be closely monitored when given bortezomib in combination with potent CYP3A4-inhibitors. Caution should be exercised when bortezomib is combined with CYP3A4- or CYP2C19 substrates.

19.2 LENALIDOMIDE

The most serious adverse reactions are venous thromboembolism (deep vein thrombosis, pulmonary embolism) and grade 4 neutropenia.

The most frequently observed adverse reactions which occurred significantly more frequently in the lenalidomide/dexamethasone group compared to the placebo/dexamethasone group were neutropenia (39.4%), fatigue (27.2%), asthenia (17.6%), constipation (23.5%), muscle cramp (20.1%), thrombocytopenia (18.4%), anaemia (17.0%), diarrhoea (14.2%) and rash (10.2%).

Myocardial Infarction: Myocardial infarction has been reported in patients receiving lenalidomide, particularly in those with known risk factors. Patients with known risk factors - including prior thrombosis - should be closely monitored, and action should be taken to try to minimize all modifiable risk factors (eg. smoking, hypertension, and hyperlipidaemia).

Venous and arterial thromboembolic events: In patients with multiple myeloma, the combination of lenalidomide with dexamethasone is associated with an increased risk of venous thromboembolism (predominantly deep vein thrombosis and pulmonary embolism) and arterial thromboembolism (predominantly myocardial infarction and cerebrovascular event). Patients and physicians are advised to be observant for the signs and symptoms of thromboembolism. Patients should be instructed to seek medical care if they develop symptoms such as shortness of breath, chest pain, arm or leg swelling.

If the patient experiences any thromboembolic events, treatment must be discontinued and standard anticoagulation therapy started. Once the patient has been stabilised on the anticoagulation treatment and any complications of the thromboembolic event have been managed, the lenalidomide treatment may be restarted at the original dose dependent upon a benefit risk assessment. The patient should continue anticoagulation therapy during the course of lenalidomide treatment.

Neutropenia and thrombocytopenia: The combination of lenalidomide with dexamethasone in multiple myeloma patients is associated with a higher incidence of grade 4 neutropenia. Patients should be advised to promptly report febrile episodes. patient management. The combination of lenalidomide with dexamethasone in multiple myeloma patients is associated with a higher incidence of grade 3 and grade 4 thrombocytopenia. Patients and physicians are advised to be observant for signs and symptoms of bleeding, including petechiae and epistaxes, especially in case of concomitant medication susceptible to induce bleeding A dose reduction of lenalidomide may be required.

A complete blood cell count, including white blood cell count with differential count, platelet count, haemoglobin, and haematocrit should be performed at baseline, every week for the first 8 weeks of lenalidomide treatment and monthly thereafter to monitor for cytopenias. The major dose limiting toxicities of lenalidomide include neutropenia and thrombocytopenia. Therefore, co-administration of lenalidomide with other myelosuppressive agents should be undertaken with caution.

Renal impairment: Lenalidomide is substantially excreted by the kidney. Therefore care should be taken in dose selection and monitoring of renal function is advised in patients with renal impairment (see section 9.2.2).

Thyroid function: Cases of hypothyroidism have been reported and monitoring of thyroid function should be considered.

Peripheral neuropathy: Lenalidomide is structurally related to thalidomide, which is known to induce severe peripheral neuropathy. At this time, the neurotoxic potential of lenalidomide associated with long-term use cannot be ruled out.

Tumour Lysis Syndrome: Because lenalidomide has anti-neoplastic activity the complications of tumour lysis syndrome may occur. The patients at risk of tumour lysis syndrome are those with high tumour burden prior to treatment. These patients should be monitored closely and appropriate precautions taken.

Allergic Reactions: Cases of allergic reaction/hypersensitivity reactions have been reported.

Severe skin reactions: Stevens-Johnson syndrome (SJS) and toxic epidermal necrolysis (TEN) have been reported. Lenalidomide must be discontinued for exfoliative or bullous rash, or if SJS or TEN is suspected, and should not be resumed following discontinuation for these reactions. Interruption or discontinuation of lenalidomide should be considered for other forms of skin reaction depending on severity.

Lactose intolerance: Revlimid capsules contain lactose. Patients with rare hereditary problems of galactose intolerance, the Lapp lactase deficiency or glucose-galactose malabsorption should not take this medicinal product.

Unused capsules: Patients should be advised never to give this medicinal product to another person and to return any unused capsules to their pharmacist at the end of the treatment.

In addition to the above adverse drug reactions identified from the pivotal trials, the following table is derived from data gathered during post-marketing studies.

System organ class	Reactions/frequency
Neoplasms benign, malignant and unspecified	Rare: Tumour lysis syndrome
Respiratory, Thoracic and Mediastinal Disorders	Unknown: Interstitial pneumonitis
Gastrointestinal disorders	Unknown: Pancreatitis
Skin and subcutaneous system disorders	Uncommon: Angioedema<br>Rare: Stevens-Johnson Syndrome^, toxic epidermal necrolysis^

19.3 DEXAMETHASONE

Allergic Reactions: Anaphylactoid reaction, anaphylaxis, angioedema.

Cardiovascular: Bradycardia, cardiac arrest, cardiac arrhythmias, cardiac enlargement, circulatory collapse, congestive heart failure, fat embolism, hypertension, hypertrophic cardiomyopathy in premature infants, myocardial rupture following recent myocardial infarction, edema, pulmonary edema, syncope, tachycardia, thromboembolism, thrombophlebitis, vasculitis.

Dermatologic: Acne, allergic dermatitis, dry scaly skin, ecchymoses and petechiae, erythema, impaired wound healing, increased sweating, rash, striae, suppression of reactions to skin tests, thin fragile skin, thinning scalp hair, urticaria.

Endocrine: Decreased carbohydrate and glucose tolerance, development of cushingoid state, hyperglycemia, glycosuria, hirsutism, hypertrichosis, increased requirements for insulin or oral hypoglycemic agents in diabetes, manifestations of latent diabetes mellitus, menstrual irregularities, secondary adrenocortical and pituitary unresponsiveness (particularly in times of stress, as in trauma, surgery, or illness), suppression of growth in pediatric patients.

Fluid and Electrolyte Disturbances: Congestive heart failure in susceptible patients, fluid retention, hypokalemic alkalosis, potassium loss, sodium retention.

Gastrointestinal: Abdominal distention, elevation in serum liver enzyme levels (usually reversible upon discontinuation), hepatomegaly, increased appetite, nausea, pancreatitis, peptic ulcer with possible perforation and hemorrhage, perforation of the small and large bowel (particularly in patients with inflammatory bowel disease), ulcerative esophagitis.

Metabolic: Negative nitrogen balance due to protein catabolism.

Musculoskeletal: Aseptic necrosis of femoral and humeral heads, loss of muscle mass, muscle weakness, osteoporosis, pathologic fracture of long bones, steroid myopathy, tendon rupture, vertebral compression fractures.

Neurological/Psychiatric: Convulsions, depression, emotional instability, euphoria, headache, increased intracranial pressure with papilledema (pseudotumor cerebri) usually after treatment, insomnia, mood swings, neuritis, neuropathy, paresthesia, personality changes, psychic disorders, vertigo.

Ophthalmic: Exophthalmos, glaucoma, increased intraocular pressure, posterior subcapsular cataracts.

Other: Abnormal fat deposits, decreased resistance to infection, hiccups, increased or decreased motility and number of spermatozoa, malaise, moon face, weight gain.

19.4 PLERIXAFOR

Adverse reactions occurring more frequently with Mozobil than placebo and considered related to Mozobil during mobilisation and apheresis in phase III studies. Very common: diarrhea, nausea, injection and infusion site reactions. Common: dizziness, headache, insomnia, gastrointestinal disorders, erythema, musculoskeletal pain, fatigue, malaise. Uncommon: allergic reaction.

20. ADVERSE EVENTS

20.1 Monitoring, Recording and Reporting of Adverse Events

An adverse event (AE) is any noxious, unintended, or untoward medical occurrence that may appear or worsen in a subject during the course of a study. It may be a new intercurrent illness, a worsening concomitant illness, an injury, or any concomitant impairment of the subject's health, including laboratory test values (as specified by the criteria below), regardless of etiology. A diagnosis or syndrome should be recorded on the AE page of the CRF rather than the individual signs or symptoms of the diagnosis or syndrome. Adverse events will be documented if observed, mentioned during open questioning, or when spontaneously reported.

Discontinuation of treatment due to either progression or deterioration of the primary malignancy should be recorded on the Treatment Discontinuation CRF as "Disease Progression" and not as an AE or an SAE.

All subjects will be monitored for AEs during the study. Assessments may include monitoring of any or all of the following parameters: the subject's clinical symptoms, laboratory, pathological, radiological or surgical findings, physical examination findings, or other appropriate tests and procedures.

All AEs will be recorded by the Investigator from the time the subject signs informed consent to 28 days after the last dose of IP. AEs and serious adverse events (SAEs) will be recorded on the

AE page of the CRF and in the subject's source documents. All SAEs must be reported to Sponsor* within 24 hours of the Investigator's knowledge of the event by facsimile, or other appropriate method, using the SAE Report Form, or approved equivalent form.

20.2 Evaluation of Adverse Events

A qualified Investigator will evaluate all adverse events as to:

20.2.1 Seriousness

A serious adverse event (SAE) is any AE occurring at any dose that:

- Results in death;
- Is life-threatening (i.e., in the opinion of the Investigator, the subject is at immediate risk of death from the AE);
- Requires inpatient hospitalization or prolongation of existing hospitalization (hospitalization is defined as an inpatient admission, regardless of length of stay);
- Results in persistent or significant disability/incapacity (a substantial disruption of the subject's ability to conduct normal life functions);
- Is a congenital anomaly/birth defect;
- Constitutes an important medical event.
- Suspected positive pregnancy

Important medical events are defined as those occurrences that may not be immediately life threatening or result in death, hospitalization, or disability, but may jeopardize the subject or require medical or surgical intervention to prevent one of the other outcomes listed above. Medical and scientific judgment should be exercised in deciding whether such an AE should be considered serious.

Second primary malignancies (SPM) will be monitored as events of interest and must be reported as serious adverse events regardless of the treatment arm the subject is in. This includes any second primary malignancy, regardless of causal relationship to study drug[s], occurring at any time for the duration of the study, from the time of signing the informed consent up to the timepoint when last study patient has been two years on maintenance and 36 months thereafter. Events of second primary malignancy are to be reported using the SAE report form and must be considered an "Important Medical Event" even if no other serious criteria apply; these events must also be documented in the appropriate page(s) of the CRF and subject's source documents. Documentation on the diagnosis of the second primary malignancy must be provided at the time of reporting as a serious adverse event (e.g., any confirmatory histology or cytology results, X-rays, CT scans, etc.).

Second primary malignancy screening includes a long term follow up period following the completion of lenalidomide treatment. Follow up of patients will be done approximately every 6 months and will be linked to the frequency of follow up within the study or to clinical practice. Follow up consists of assessment of status and incidence of any SPMs.

Events **not considered** to be SAEs are hospitalizations which:

- Routine treatment or monitoring of the studied indication not associated with any deterioration in condition.
- A procedure that was planned before entry into the clinical study
- An elective treatment of a pre-existing condition unrelated to the studied indication or its treatment
- Emergency outpatient treatment or observation that does not result in admission, unless fulfilling other seriousness criteria above.

If an AE is considered serious, both the AE page/screen of the CRF and the SAE Report Form must be completed.

For each SAE, the Investigator will provide information on severity, start and stop dates, relationship to study drug, action taken regarding study drug, and outcome.

20.2.2 Severity / Intensity

For both AEs and SAEs, the Investigator must assess the severity / intensity of the event.

The severity / intensity of AEs will be graded on a scale of 1 to 5 according to the National Cancer Institute (NCI) Common Terminology Criteria for Adverse Events Version 4.03 (NCI CTCAE): http://ctep.cancer.gov/reporting/ctc.htlm.

AEs that are not defined in the NCI CTCAE should be evaluated for severity / intensity according to the following scale:

- Grade 1 = Mild – asymptomatic or mild symptoms; clinical or diagnostic observations only: intervention not indicated
- Grade 2 = Moderate – minimal, local or non-invasive intervention indicated; limiting age-appropriate instrumental activities of daily living (ADL)
- Grade 3 = Severe or medically significant but not immediately life-threatening: hospitalization or prolongation of hospitalization indicated; disabling; limiting self care ADL
- Grade 4 = Life threatening consequences; urgent intervention indicated
- Grade 5 = Death related to AE

Instrumental ADL refer to preparing meals, using the telephone etc. Self care ADL refer to bathing, dressing, using the toilet etc.

20.2.3 Classification of relationship/causality of adverse events (SAE/AE) to study medication

20.2.4 Causality assessment

The investigator must determine the relationship between the administration of study drugs and the occurrence of an AE/SAE as Not Suspected or Suspected as defined below:

Not suspected:	The temporal relationship of the adverse event to IP administration makes **a causal relationship unlikely or remote**, or other medications, therapeutic interventions, or underlying conditions provide a sufficient explanation for the observed event.
Suspected:	The temporal relationship of the adverse event to IP administration makes **a causal relationship possible**, and other medications, therapeutic interventions, or underlying conditions do not provide a sufficient explanation for the observed event.

20.2.5 Duration

For both AEs and SAEs, the Investigator will provide a record of the start and stop dates of the event.

20.2.6 Action Taken

The Investigator will report the action taken with study drug as a result of an AE or SAE, as applicable (e.g., discontinuation or reduction of study drug, as appropriate) and report if concomitant and/or additional treatments were given for the event.

20.2.7 Outcome

The investigator will report the outcome of the event for both AEs and SAEs.

AEs that cause a subject to discontinue study participation must be followed until either the event resolves, stabilizes or returns to baseline (if a baseline assessment is available). All SAEs that have not resolved upon discontinuation of the subject's participation in the study must be followed until recovered, recovered with sequelae, not recovered (death due to another cause) or death (due to the SAE).

20.3 Abnormal Laboratory Values

An abnormal laboratory value is considered to be an AE if the abnormality:

- results in discontinuation from the study;
- requires treatment, modification/ interruption of study drug dose, or any other therapeutic intervention; or
- is judged to be of significant clinical importance.

Regardless of severity grade, only laboratory abnormalities that fulfill a seriousness criterion need to be documented as a serious adverse event.

If a laboratory abnormality is one component of a diagnosis or syndrome, then only the diagnosis or syndrome should be recorded on the AE page/screen of the CRF. If the abnormality was not a part of a diagnosis or syndrome, then the laboratory abnormality should be recorded as the AE.

20.4 Suspected unexpected serious adverse reaction (SUSAR)

The investigator-sponsor will inform Health Authorities and Ethical Committee of adverse drug reactions that are serious, unlisted/unexpected and that have not previously been reported in the investigators brochure or reference safety information document. A clear description of the suspected reaction should be provided along with an assessment as to whether the event is drug or disease related. Any suspected unexpected serious adverse reactions (SUSARs) arising from this trial will be reported to the investigators to company, and to all applicable Ethics Committees and Health Authorities within the timelines required by the EU Clinical Trial Directive. The manner of SUSAR reporting will be in compliance with the procedures of the Ethics Committees and Health Authorities involved.

20.5 Pregnancy

This study will follow the Pregnancy Prevention Risk Management Plan which includes three parts:

1) Lenalidomide Risks of Fetal Exposure Pregnancy Testing Guidelines and Acceptable Birth Control Methods
2) Lenalidomide Education and Counseling Guidance Document for Investigator
3) Lenalidomide Information Sheet for Patients

Pregnancies occurring while subjects are on lenalidomide or within 28 days after a subject's last dose of lenalidomide are considered events to be reported immediately to Celgene. If the subject is on study drug the study drug is to be discontinued immediately and the subject is to be instructed to return any unused portion of the study drug to the investigator. The pregnancy must be reported to Celgene immediately of the Investigator's knowledge of the pregnancy by phone and facsimile using the SAE Form. The pregnancy must also be reported to the sponsor. The investigator will follow the subject until completion of the pregnancy, and must notify the sponsor and Celgene of the outcome immediately or as specified below. The investigator will provide this information as a follow-up to the initial pregnancy report.
If the outcome of the pregnancy meets the criteria for immediate classification as a SAE (i.e., spontaneous abortion [any congenital anomaly detected in an aborted foetus is to be Documented), stillbirth, neonatal death, or congenital anomaly), the investigator should follow the procedures for reporting SAEs. All neonatal deaths that occur within 28 days of birth should be reported, without regard to causality, as SAEs. In addition, any infant death after 28 days that the investigator suspect is related to the in utero exposure to lenalidomide should also be reported.
In the case of a live "normal" birth, Celgene should be advised as soon as the information is available.
Any suspected foetal exposure to lenalidomide must be reported to Celgene within 24 hours of being made aware of the event. The female subject should be referred to an obstetrician/gynecologist experienced in reproductive toxicity for further evaluation and counseling.
If a female partner of a male subject taking investigational product becomes pregnant, the male subject taking product should notify the investigator, and the pregnant female partner should be advised to call their healthcare provider immediately.

If a pregnancy related event is reported in a female partner of a male subject, the investigator should ask if the female partner is willing to share information with Celgene Drug Safety and allow the pregnancy related event to be followed up to completion.

20.6 Expedited Reporting of Adverse Events

20.6.1 Reporting to Regulatory Authorities and the Ethics Committee

The Sponsor will inform relevant Regulatory Authorities and Ethics Committees;

- Of all relevant information about serious unexpected adverse events suspected to be related to the IP that are fatal or life-threatening as soon as possible, and in any case no later than seven days after knowledge of such a case. Relevant follow-up information for these cases will be subsequently be submitted within an additional eight days
- Of all other serious unexpected events suspected to be related to the IP as soon as possible, but within a maximum of fifteen days of first knowledge by the investigator.

20.6.2 Immediate reporting by Investigator to Sponsor and Celgene

The investigator will inform the sponsor and Celgene of any serious adverse event. This applies to all SAEs, regardless of relationship to the study medication, that occur during the study, those made known to the investigator within 28 days after a subject´s last dose of study drug, and those made known to the investigator at any time that are suspected of being related to the study medication. This must be documented on an SAE form. This form must be completed in English and supplied to Celgene within 24 hours. The initial report must be as complete as possible, including details of the current illness and serious adverse event, and an assessment of the causal relationship between the event and the investigational product. Information not available at the time of the initial report (e.g. an end date for the AE or laboratory values received after the report) must be documented on a SAE form.

The Sponsor will provide Celgene with a copy of the annual safety report at the time of submission to the regulatory authority and Ethics Committee.

Contact details for Sponsor:
Kuopio University Hospital
Seppo Lehto
Clinic of Internal Medicine/Center of Medicine
PB 1777
FI-70 211 Kuopio, Finland
Tel: +358 17 17 2161
Fax: +358 17 173 993
E-mail: seppo.lehto@kuh.fi

Contact details for Drug Safety Celgene Nordics:
Celgene AB
Kista Science Tower
164 51 Kista, Sweden
Phone: +46 8 703 16 31
Fax: +46 8 703 16 03
E-mail: drugsafety-nordic@celgene.com

21. ADVERSE EVENT UPDATES

Celgene shall notify the principle investigator/sponsor-investigator of the following information:
1. Any AE associated with the use of study drug or in other studies that is both serious and unexpected.
2. Any finding from tests in laboratory animals that suggests a significant risk for human subjects including reports of mutagenicity, teratogenicity, or carcinogenicity.

The principle investigator/sponsor will forward this information to other investigators involved in the trial. The sponsor-investigator shall notify the EC and the relevant regulatory authorities of any new significant risks to subjects as required. The local Ethics Committee (EC) must be informed by the principal investigator about local SAEs according to the regulations according to the EU Clinical Directive2001/20/EC and/or national law. All adverse events occurring during the treatment period and until the end of the last treatment administration will be reported in the treatment evaluation form.

22. SUSARs

Forms for reporting SUSAR´s to Health Authorities are available at the website of the Finnish Medicines Agency, www.fimea.fi.

Reporting of Suspected Unexpected Serious Adverse Reactions (SUSARs), will be collected and reported to the competent authorities and relevant ethics committees in accordance with Directive 2001/20/EC or as per national regulatory requirements in participating countries.

23. ANNUAL SAFETY REPORT

The sponsor will submit annual safety report to the Ethics Committee and Health Authorities (FIMEA) and to Celgene.

24. DATA SAFETY AND MONITORING BOARD

A data and safety monitoring board will be installed before start of the study. Serious adverse event reporting will be evaluated twice a year in the meetings of Finnish Leukemia and Myeloma Group. Monitoring of the study will be arranged at the level of grade 1-2.

25. CASE REPORT FORMS

Data will be reported on electronic Case Report Forms (CRF) and the CRF registry is part of official and licenced Finnish Hematology Register. Data will be collected to document eligibility, safety and efficacy parameters and it will include at least inclusion and exclusion criteria, baseline medical history and status, stage of disease, timing and dosage of treatment, adverse events, parameters to evaluate the study endpoints, reason for end of protocol treatment and survival status of patient. All CRF entries must be based on source documents.

26. STATISTICAL CONSIDERATIONS

This is a phase II study, which is efficacy, safety and dose-finding study. We are looking for the efficient and safe administration of VRD-induction before ASCT and post-ASCT lenalidomide maintenance. In the historical, national, phase II, Finnish Vel-Dex study we have included 47 patients. Total patient sample size of this new trial is 80; the reasonable number of study patients in a phase II trials is considered to be between 40 – 100 patients.

So far the immunophenotypic remission rate of patients in ongoing Vel-Dex trial is 38% 3 months after ASCT(35). The estimation of immunophenotypic remission rate of this trial at the same time point is 40 – 45%. The largest estimate for immunophenotypic remission after lenalidomide maintenance would be 55%.

The immunophenotypic rate will be considered as primary endpoint for the sample size calculation.

- Let P0 be the largest immunophenotypic remission rate which, if true, implies that the therapeutic activity is too low and therefore does not warrant further investigation. In the present trial, P0 has been taken as 38 %.
- Let P1 be the smallest immunophenotypic remission rate which, if true, implies that the therapeutic activity is sufficiently high and therefore the protocol treatment warrants further investigation in clinical trials. In the present trial, P1 has been taken as 55%.

```
running N:\Stata8\profile.do ...

. sampsi 0.38 0.55, power(0.8) onesample

Estimated sample size for one-sample comparison of proportion
  to hypothesized value

Test Ho: p = 0.3800, where p is the proportion in the population

Assumptions:

         alpha =   0.0500  (two-sided)
         power =   0.8000
 alternative p =   0.5500

Estimated required sample size:

             n =       65
```

However, in order to overcome dropout (15-20%), 80 patients will be included in this phase II trial.

Interim analysis will be performed after 30 patients have been achieved time point 3 months post-ASCT, to assess the success of study treatment before maintenance. Administration of lenalidomide is different in this trial as compared with other studies regarding lenalidomide consolidation and maintenance (30,31). During lenalidomide maintenance improvement of responses and PFS will be assessed and compared to no maintenance (as in earlier Vel-Dex study).

Analysis plan:

- Comparison of response rates and especially immunophenotypic (molecular response) data with Finnish historical Vel-Dex study (assessment at identical time points).
- Comparison of PFS and OS with the same study
- Correlation of patient characteristics, including cytogenetic data at diagnosis, to different responses achieved

<u>Efficacy analyses</u>

The main endpoint of this phase II is the proportion of patients who obtain an immunophenotypic remission during protocol treatment. A 95% confidence interval (CI) will be constructed, and the null hypothesis (38 %) will be rejected in favour of the alternative hypothesis (55 %), if the lower bound of the 95% CI is larger than 0.38. Efficacy analyses will be intention-to-treat and included.

In the mobilization randomization substudy 40 patients per arm will be sufficient to assess if there are essential differences between the number of collection days, CD34+ cell yields and in-hospital days. No formal interim analysis is planned regarding efficacy.

27. ETHICS

National Ethics Committee and Finnish Medicines Agency will approve the study protocol and any substantial amendment. In accordance with the Declaration of Helsinki/Tokyo/Venice (28) patients have the right to withdraw from the protocol at any time for any reason. The investigator also has the right to withdraw patients from the protocol in the event of intercurrent illness, adverse events and treatment failure after a prescribed procedure, protocol violations, cure, administrative reasons or other reasons. If a patient decides to withdraw from the protocol, all efforts will be made to complete and report the observations as thoroughly as possible. A complete final evaluation at the time of the patient's withdrawal should be made together with the reason. The clinical trial investigator-sponsor is the physical person or legal entity which is interested in the performance of the trial, signs requests for authorization addressed to the Ethics Committees (ECs) and regulatory authorities in respective countries and is responsible for the trial, including its performance, initiation and completion. The sponsor will be responsible for ensuring compliance with applicable legal guidelines.
Investigator must agree with this protocol and know in detail the properties of the drug used in this clinical trial. Investigator must provide the patient with a patient information sheet and help him/her to understand the explanation provided. It is important to tell the patient that his/her participation in the study is completely voluntary and that it will not affect patient-physician relationship. In addition, it will be guaranteed that all people involved in the study will observe the confidentiality of any information related to the patient. The investigator is also responsible for ensuring that the clinical study is performed in accordance with the protocol, current guidelines on Good Clinical Practice (GCP, ICH/135/95), EU-directive (2001/20/EC) and applicable regulatory requirements. All participants in the study are covered by the National Pharmaceutical Insurance Pool.
The local investigator is responsible for the proper conduct of the study at the study site.

28. PATIENT INFORMATION AND CONSENT

Written informed consent of patient is required before any study related procedure. The investigator should provide enough time for patient to discuss about all details of the study. All questions concerning the study will be answered to the satisfaction of the patient before possible obtaining of consent. The content of the patient information letter, informed consent form and any other written information provided to patients will be in compliance with ICH-GCP and other applicable regulations and should be approved by the Ethics Committee before use. Whenever new important information, relevant to the patients´s consent, will be available, the patient information letter, informed consent and any other written information will be revised. Any revised

informed consent form and written information should be approved by the Ethics Committee before use. The patient should be informed in a timely manner if new information becomes available that might be relevant to the patients´s willingness to continue participation in the study.

29. PATIENT CONFIDENTIALITY

Each patient is assigned a unique patient study number at registration. In study documents the patient´s identity is coded by patient study number. In some cases date of birth is also listed. The local investigator will keep a subject enrolment and identification log that contains the key to the code, the personal identification data linked to each patient study number. This data is filed at the investigational site and should only be accessed by the investigator and the supporting site staff or by representatives of the sponsor-investigator or a regulatory agency for the purpose of monitoring visits or audits and inspections. The Information and Consent Form also explains that for data verification purposes an authorized regulatory authority, or an ethics review board may require direct access to parts of the hospital or practice records relevant to the study including patients' medical history.

30. STUDY INSURANCE

Before the start of the study the sponsor-investigator will ensure that adequate insurance for patients is in place covering losses due to death or injury resulting from the study, in accordance with applicable laws and regulations. Adequate insurance for investigators will be ensured.

31. MONITORING

Independent staff from another institution or CRO company, not involved in the study, will perform monitoring of the study. Inclusion criteria, endpoints and all key test results according to the assessment schedule will be monitored to assure data quality. The monitoring will take place at least at that time when the patient has completed the study.

The investigator is required to permit direct access to the facilities where the study took place, source documents, CRFs and applicable supporting records of subject participation for audits and inspections by Ethics Committees and National Medicines Agency. The investigator should make every effort to be available for the audits and/or inspections.

32. DATA AND DOCUMENTS HANDLING

Documents which are essential for evaluation of conduct of the study and the quality of data will be filed in such a manner that they are protected from accidental loss. The sponsor-investigator will file all national essential regulatory documents relevant to the overall conduct of the trial. Local investigators will file all essential documents relevant to the conduct of the trial on site. Essential documents will be retained for at least 10 years after the end of the trial and the final

presentation of the study. Source documents and medical records of patients should be retained for at least 10 years after the end of the trial. After this time these documents will be handled by the site´s guidelines regarding medical records.

33. STORAGE OF SAMPLES

Biological samples should only be stored for the purpose of additional research if the patent has given consent. If no informed consent was obtained, samples should be destroyed after the patient has completed all protocol treatment and procedures.

34. AMENDMENTS

Any amendments to this protocol that seems appropriate, as the study proceeds (regarding safety or efficacy) will be agreed upon the coordinating and/or principal investigator. Amendments will be submitted to the Ethics Committee and the regulatory authority for written approval before the implementation of the amended version.

35. ANNUAL REPORTING AND UPDATING

The sponsor will submit a summary of the progress of the trial to the accredited Ethics Committee once a year. Information will be provided on the date of inclusion of the first patient, numbers of patients included and numbers of patients that have completed the trial, serious adverse events/serious adverse reactions, other problems, and amendments.

36. PUBLICATION POLICY

Study will be registrated in clinicaltrials.gov registry. The final publication of the study results will be written by the principal investigator, co-investigators and the cooperative group, the Finnish Myeloma Study Group. At least one representative from each center who has included patients will be named in the list of authors. Several publications are planned; CR rate, feasibility, survival, mobilization study and correlative immunological/biological study. Study results will be submitted for publication in a peer reviewed scientific journal regardless of the outcome of the study. The ownership of the data generated in this study belongs to the Investigator-Sponsor. Celgene will be able to review the manuscript at least 30 days before submission.

37. FLOW CYTOMETRY PROTOCOL

will be updated in May 2012 in annual meeting of Finnish laboratory hematologist in Helsinki.

38. INSTRUCTIONS FOR DNA SAMPLES AND BIOBANKING

The Finnish Hematology Register and Biobank is a new national project which will probably cover all haematological units in years 2012-2014. A written informed consent will be required.

Every transfer of material (collection, processing and shipping) must be locally documented. All biobanking samples will have the same code as the study patient identification code. Information regarding the material collected, date and time of collection as well as date and time of processing will be recorded. The patient retains the right to have the sample material destroyed at any time by contacting Principal Investigator. However, already obtained data from this material can be used for intended analysis. This comprehensive biobank project will be used also in this study.

Instructions for biobanking samples are available on www.hematology.fi/biobank.

Samples send to TYKSLAB, Department of Molecular Genetics for PCR-analysis will be handled as routine clinical samples by patient social security number and name. Instructions see page 31.

39. STUDY COMPLETION AND TERMINATION

The study will be considered complete when all subjects have completed the final assessment and the follow up assessment. This study may be prematurely terminated if, in investigator-sponsor's opinion, there is a reasonable reason. Investigator will receive a written notification in which the terminating party documents the reason for study termination. Circumstances justifying study termination include, but are not limited to:

- Unforeseen, considerable and unacceptable risks for patients.
- Impossibility to recruit an acceptable number of patients
- Failure to adhere to protocol requirements

40. FINANCING

This study of Finnish Myeloma Group has received a grant of 200 000E from Celgene Corporation. This grant will cover the monitoring and extra costs; minimal residual disease assessment by ASO-PCR if the patient has achieved immunophenotypic remission. Celgene Corporation will supply the study drug, lenalidomide, which is the investigational product and is not yet approved for first-line treatment or maintenance in multiple myeloma. Additional applications to cover the costs of the stem cell the mobilization substudy and graft analysis have been sent.

APPENDIX 1 / IMWG criterias

1. International Staging System for Multiple Myeloma (ISS stage)

International Staging System for Multiple Myeloma of the International Myeloma Working Group (J Clin Oncol 2005: 23; 3412-3420).

Stage	Criteria
I	Serum β2-microglobulin < 3.5 mg/L<br>Serum albumin ≥ 3.5 g/dL
II	Neither stage I nor stage III*
III	Serum β2-microglobulin ≥ 5.5 mg/L

* There are two categories for stage II: serum β2-microglobulin < 3.5 mg/L but serum albumin < 3.5 g/dL; or serum β2- microglobulin 3.5 to < 5.5 mg/L irrespective of the serum albumin level.

2. Diagnostic criteria for multiple myeloma requiring systemic therapy

Presence of an M-component in serum and/or urine plus clonal plasma cells in the bone marrow and/or a documented clonal plasmacytoma

And one or more of the following:

Calcium elevation (> 2.65 mmol/L)
Renal insufficiency (creatinine > 2 mg/dL = >173µmol/l)
Anemia (hemoglobin < 10 g/dL or 2 g/dL below normal)
Bone disease (lytic lesions or osteopenia)

3. International uniform response criteria for multiple myeloma

Based on International Myeloma Working Group uniform response criteria. B.G.M. Durie et al. (Leukemia, 2006: 20; 1467-1473)

RESPONSE CRITERIA:
NOTE: Once (s)CR is established, response remains (s)CR until relapse is documented.

Response subcategory	*Response criteria*[a]
sCR*	CR as defined below plus<br>▪ Normal FLC ratio and<br>▪ Absence of clonal cells in bone marrow[b] by immunohistochemistry or immunophenotyping[c]
CR	▪ Negative immunofixation on the serum and urine and<br>▪ Disappearance of any soft tissue plasmacytomas and<br>▪ ≤ 5% plasma cells in bone marrow[b]
VGPR	Serum and urine M-protein detectable by immunofixation but not on electrophoresis or 90% or greater reduction in serum M-protein plus urine M-protein level < 100 mg per 24 h
PR	▪ ≥ 50% reduction of serum M-protein and reduction in 24-h urinary M-protein by ≥ 90% or to < 200 mg per 24 h<br>▪ In addition to the above listed criteria, if present at baseline, a ≥ 50% reduction in the size of soft tissue plasmacytomas is also required
SD[d]	Not meeting criteria for CR, VGPR, PR or progressive disease

* will only be determined in case the FLC assay is available in the participating hospitals

Abbreviations: CR, complete response; FLC, free light chain; PR, partial response; SD, stable disease; sCR, stringent complete response; VGPR, very good partial response. a All response categories require two consecutive assessments made at anytime before the institution of any new therapy; all categories also require no known evidence of progressive or new bone lesions if radiographic studies were performed.
b Confirmation with repeat bone marrow examination not needed. c Presence/absence of clonal cells is based upon the κ/λ ratio. An abnormal k/l ratio by immunohistochemistry and/or immunofluorescence requires a minimum of 100 plasma cells for analysis. An abnormal ratio reflecting presence of an abnormal clone is κ/λ of > 4:1 or < 1:2. d not recommended for use as an indicator of response; stability of disease is best described by providing the time to progression estimates

Kyle RA, Rajkumar SV. Criteria for diagnosis, staging, risk stratification and response assessment of multiple myeloma. Leukemia 2009;23:3-9.

Table 5 International Myeloma Working Group uniform response criteria for multiple myeloma[29]

Response subcategory	*Response criteria*
Complete response[a] (CR)	Negative immunofixation of serum and urine and<br>Disappearance of any soft tissue plasmacytomas, and<br>$<$5% plasma cells in bone marrow
Stringent complete response (sCR)	CR as defined above plus<br>Normal FLC ratio and<br>Absence of clonal cells in bone marrow by immunohistochemistry or immunofluorescence
Very good partial response (VGPR)[a]	Serum and urine M-component detectable by immunofixation but not on electrophoresis or<br>$\geqslant$90% or greater reduction in serum M-component plus urine M-component $<$100 mg per 24 h
Partial response (PR)	$\geqslant$50% reduction of serum M protein and reduction in 24-h urinary M protein by $\geqslant$90% or to $<$200 mg per 24 h<br>If the serum and urine M protein are unmeasurable, a $\geqslant$50% decrease in the difference between involved and uninvolved FLC levels is required in place of the M protein criteria<br>If serum and urine M protein are unmeasurable, and serum free light assay is also unmeasurable, $\geqslant$50% reduction in bone marrow plasma cells is required in place of M protein, provided baseline percentage was $\geqslant$30%<br>In addition to the above criteria, if present at baseline, $\geqslant$50% reduction in the size of soft tissue plasmacytomas is also required
Stable disease (SD)	Not meeting criteria for CR, VGPR, PR or progressive disease
Progressive disease (PD)[a]	Increase of 25% from lowest response value in any one or more of the following:<br>Serum M-component (absolute increase must be $\geqslant$0.5 g/100 ml)[c] and/or<br>Urine M-component (absolute increase must be $\geqslant$200 mg per 24 h) and/or<br>Only in patients without measurable serum and urine M-protein levels: the difference between involved and uninvolved FLC levels (absolute increase must be $>$100 mg/l)<br>Bone marrow plasma cell percentage (absolute % must be $\geqslant$10%)<br>Definite development of new bone lesions or soft tissue plasmacytomas or definite increase in the size of existing bone lesions or soft tissue plasmacytomas<br>Development of hypercalcemia (corrected serum calcium $>$11.5 mg/100 ml) that can be attributed solely to the plasma cell proliferative disorder

[a]Note clarification to IMWG criteria for coding CR and VGPR in patients in whom the only measurable disease is by serum FLC levels: CR in such patients is defined as a normal FLC ratio of 0.26–1.65 in addition to CR criteria listed above. VGPR in such patients is defined as a $>$90% decrease in the difference between involved and uninvolved free light chain (FLC) levels.
All response categories (CR, sCR, VGPR and PR) require two consecutive assessments made at any time before the institution of any new therapy; complete, PR and SD categories also require no known evidence of progressive or new bone lesions if radiographic studies were performed. Radiographic studies are not required to satisfy these response requirements. Bone marrow assessments need not be confirmed.
Adapted with permission from Durie *et al.* [29]
c for progressive disease, serum M-component increases of $\geqslant$1 gm/100 ml are sufficient to define relapse if starting M-component is $\geqslant$5 gm/100 ml.

RELAPSE CRITERIA:

Relapse subcategory	*Relapse criteria*
Progressive disease[a]<br><br>To be used for calculation of time to progression and progression-free survival end points for all patients including those in CR (includes primary progressive disease and disease progression on or off therapy)	Progressive Disease: requires any one or more of the following:<br><br>▪ Increase of ≥ 25% from lowest response level in serum M-component (the absolute increase must be ≥ 0.5 g/dl)[b] and/or<br>▪ Increase of ≥ 25% from lowest response level in urine M-component (the absolute increase must be ≥ 200 mg/24 h) and/or<br>▪ Increase of ≥ 25% from lowest response level in bone marrow plasma cell percentage: the absolute % must be ≥10%[c]<br>▪ Definite development of new bone lesions or soft tissue plasmacytomas or definite increase in the size of existing bone lesions or soft tissue plasmacytomas<br>▪ Development of hypercalcaemia (corrected serum calcium > 11.5 mg/dl or 2.65 mmol/l) that can be attributed solely to the plasma cell proliferative disorder
Clinical relapse[a]	Clinical relapse requires one or more of:<br><br>Direct indicators of increasing disease and/or end organ dysfunction (CRAB features)[b]. It is not used in calculation of time to progression or progression-free survival but is listed here as something that can be reported optionally or for use in clinical practice<br>1. Development of new soft tissue plasmacytomas or bone lesions<br>2. Definite increase in the size of existing plasmacytomas or bone lesions. A definite increase is defined as a 50% (and at least 1 cm) increase as measured serially by the sum of the products of the cross-diameters of the measurable lesion<br>3. Hypercalcaemia (> 2.65 mmol/l) [11.5 mg/dl]<br>4. Decrease in hemoglobin of ≥ 1.25 mmol/l [2 g/dl]<br>5. Rise in serum creatinine by 177 μmol/l or more [2 mg/dl or more]
Relapse from CR[a]<br><br>(To be used only if the end point studied is DFS)[d]	Any one or more of the following:<br>▪ Reappearance of serum or urine M-protein by immunofixation or electrophoresis<br>▪ Development of ≥ 5% plasma cells in the bone marrow[c]<br>▪ Appearance of any other sign of progression (i.e., new plasmacytoma, lytic bone lesion, or hypocalcaemia see above)

Abbreviations: CR, complete response; DFS, disease-free survival. a All relapse categories require two consecutive assessments made at anytime before classification as relapse or disease progression and/or the institution of any new therapy. b For progressive disease, serum M-component increases of ≥ 10 g/l are sufficient to define relapse if M-component is ≥ 50 g/l. c Relapse from CR has the 5% cutoff versus 10% for other categories of relapse. d For purposes of calculating time to progression and progression-free survival, CR patients should also be evaluated using criteria listed above for progressive disease

APPENDIX 2: WHO /NYHA

UBROD-ECOG-WHO Performance Status Scale

0	Normal activity
1	Symptoms, but nearly ambulatory
2	Some bed time, but to be in bed less than 50% of normal daytime
3	Needs to be in bed more than 50% of normal daytime
4	Unable to get out of bed

NYHA* scoring list

Grade 1	No breathlessness
Grade 2	Breathlessness on severe exertion
Grade 3	Breathlessness on mild exertion
Grade 4	Breathlessness at rest

The *New York Heart Association functional and therapeutic classification applied to dyspnoea

APPENDIX 3

F. Management of patients with Bortezomib (Velcade®)-related neuropathic pain and/or peripheral sensory neuropathy

		Peripheral Sensory Neuropathy (NCI CTC Grade)				
Neuropathic Pain (NCI CTC Grade)		0	1	2	3	4
		Normal	Asymptomatic; loss of deep tendon reflexes or paresthesia (including tingling) but not interfering with function	Sensory alteration or paresthesia (including tingling), interfering with function, but not interfering with ADL	Sensory alteration or paresthesia interfering with ADL	Disabling
0	None	No action	No action	Reduce to once weekly or 25% dose reduction	Hold; 50% dose reduction; Schedule Δ required	Discontinue Bortezomib
1	Mild pain not interfering with function	No action	Reduce to once weekly	25% dose reduction	Hold; 50% dose reduction; Schedule Δ required	Discontinue Bortezomib
2	Moderate pain: pain or analgesics interfering with function, but not daily activities	25% dose reduction	50% dose reduction	Hold; 50% dose reduction	Hold; 50% dose reduction; schedule Δ required	Discontinue Bortezomib
3	Severe pain: pain or analgesics severely interfering with daily activities	Hold; 50% dose reduction; Schedule Δ required	Hold; 50% dose reduction; schedule Δ required	Hold; 50% dose reduction; schedule Δ required	Discontinue Bortezomib	Discontinue Bortezomib
4	Disabling	Discontinue Bortezomib	Discontinue Bortezomib	Discontinue Bortezomib	Discontinue Bortezomib	Discontinue Bortezomib

Key:

Hold:	Interrupt Bortezomib for up to 2 weeks until the toxicity returns to Grade 1 or better.
25% Dose reduction:	Bortezomib dose reduction from 1.3 to 1.0 mg/m^2/dose.
50% Dose reduction:	Bortezomib dose reduction from 1.3 to 0.7 mg/m^2/dose.
Schedule Δ required:	Schedule change from Bortezomib twice per week (Days 1, 4, 8 and 11) to once per week (Days 1, 8, 15, and 22) required. If the patient is already on a once weekly schedule, then the drug will be given every other week (e.g. Day 1, Day 15)

References:

1. Bird JM, Owen RG, D´Sa S et al. Guidelines for the diagnosis and management of multiple myeloma. Br J Haemat 2011;154:32-75
2. Van de Velde HJK, Liu X, Chen G, Cakana A, Draedt W, Bayssas M. Complete response correlates with long-term survival and progression-free survival in high-dose therapy in multiple myeloma. Haematologica 2007;92:1399–1406.
3. Corradini P, Voena C, Astolfi M et al. High-dose sequential chemoradiotherapy in multiple myeloma: Residual tumor cells are detectable in bone marrow and peripheral blood cell harvests and after autografting. Blood 1995;85:1596-1602
4. Corradini P, Voena C, Tarella C et al. Molecular and clinical remissions in multiple myeloma: Role of autologous and allogeneic transplantation of hematopoietic cells. J Clin Oncol 1999;17:208-215
5. Corradini P, Cavo M, Lokhorst H et al. Molecular remission after myeloablative allogeneic stem cell transplantation predicts a better relapse-free survival in patients with multiple myeloma, Blood 2003;102:1927-1929
6. Bakkus MH, Bouko Y, Samson D et al. Post-transplantation tumour load in bone marrow, as assessed by quantitative ASO-PCR, is a prognostic parameter in multiple myeloma. Br J Haematol 2004;126:665-674
7. Galimberti S, Benedett E, Morabito F et al. Prognostic role of minimal residual disease in multiple myeloma patients after non-myeloablative allogeneic transplantation. Leuk Res 2005;29:688-693
8. Kröger N, Zagrivnaja M, Schwartz S et al. Kinetics of plasma-cell chimerism after allogeneic stem cell transplantation by highly sensitive real-time PCR based on sequence polymorphism and its value to quantify minimal residual disease in patients with multiple myeloma. Exp Hematol 2006;34:688-693
9. Tricot GJ. What is the significance of molecular remission in multiple myeloma? Clin Adv Hematol & Oncol 2007;5:91-95
10. Ladetto M, Pagliano G, Ferrero S et al. Major tumor shrinking and persistent molecular remissions after consolidation with bortezomib, thalidomide and dexamethasone in patients with autografted myeloma. J Clin Oncol 2010;28:2077-2084
11. Putkonen M, Kairisto V, Juvonen V et al. Depth of response assessed by quantitative ASO-PCR predicts the outcome after stem cell transplantation in multiple myeloma. Eur J Haematol 2010;85:416-423
12. Ladetto M, Ferrero S, Drandi D et al. Long-term results of the GIMEMA VTD Consolidation trial in autografted multiple myeloma patients(VEL-03-096): Impact of minimal residual detection by real time quantitative PCR on late recurrences and overall survival.
    Abstract#827 ASH 2011
13. Sarasquete ME, Carcia-Sanz R, Gonzales D et al. Minimal residual disease monitoring in multiple myeloma: a comparison between allelic-spesific oligonucleotide real-time quantitative polymerase chain reaction and flow cytometry. Haematologica 2005;90:1365-1372
14. Paiva B, Vidriales MB, Cervero J et al. Multiparameter flow cytometric remission is the most relevant prognostic factor for multiple myeloma patients who undergo autologous stem cell transplantation. Blood 2008;112:4017-4023
15. Rawstron AC, Orfao A, Beksac M et al. Report of the European Myeloma Network on multiparametric flow cytometry in multiple myeloma and related disorders. Haematologica 2008;93:431-438
16. Rawstron A, De Tute R, Child A et al. Minimal residual disease (MRD) assessment using multiparameter flow cytometry (MFC) predicts outcome in both intensively and non-intensively treated patients: Results from the MRC Myeloma IX Trial et al. Haematologica 2011;96 Suppl 1:S1-S178. Abstract 0-09.
17. Richardson PG, Barlogie B, Berenson J, et al. A phase 2 study of bortezomib in relapsed, refractory myeloma [see comment]. N Engl J Med 2003;348:2609–2617.
18. Jagannath S, Barlogie B, Berenson J, et al. A phase 2 study of two doses of bortezomib in relapsed or refractory myeloma. Br J Haematol 2004;127:165–172.

19. Richardson PG, Sonneveld P, Schuster MW, et al. Bortezomib or high-dose dexamethasone for relapsed multiple myeloma [see comment]. N Engl J Med 2005;352:2487–2498.
20. Harousseau JL, Attal M, Leleu X, et al. Bortezomib plus dexamethasone as induction treatment prior to autologous stem cell transplantation in patients with newly diagnosed multiple myeloma: results of an IFM phase II study. Haematologica 2006; 91:1498–1505.
21. Harousseau JL, Attal M, Loiseau HA et al. Bortezomib plus dexamethasone is superior to vincristine plus doxorubicin plus dexamethasone as induction treatment prior to autologous stem-cell transplantation in newly diagnosed multiple myeloma: Results of the IFM 2005-01 phase III trial. J Clin Oncol 2010;28:4621-4629
22. Cavo M, Tacchetti P, Patriarca F et al. Bortezomib with thalidomide puls dexamethasone compared with thalidomide plus dexamethasone as induction therapy before, and consolidation therapy after double autologous stem cell transplantation in newly diagnosed multiple myeloma: a randomized phase 3 study. Lancet 2010;376:2075-2085
23. Sonneveld P, van der Holt B, Schmidt-Wolf IGH et al. First analysis of HOVON-65/GMMG-HD4 randomized phase III trial comparing bortezomib, adriamycine, dexamethasone (PAD) vs VAD as induction tratment prior to high dose melphalan (HDM) in patients with newly diagnosed multiple myeloma (MM). Blood 2008 (ASH Annual Meeting Abstracts), 112:Abstract 653
24. Rosinol L , Cibeira MT, Martinez J et al. Thalidomide/dexamethasone (TD) vs bortezomib (velcade/thalidomide/dexamethasone (VTD) vs VBMCP/VBAD/velcade as induction regimens prior autologous stem cell transplantation (ASCT) in multiple myeloma (MM): Results of a phase III PETHEMA/GEM trial. Blood (ASH Annual Meeting Abstracts) 2009;114, Abstract 130
25. Terragna C, Zamagni E, Petrucci MT et al. Molecular remission after bortezomib-thalidomide-dexamethasone compared with thalidomide-dexamethasone as consolidation therapy following double autologous transplantation for multiple myeloma: Results of a qualitative and quantitative analysis.Blood (ASH Annual Meeting Abstracts) 2010;116:Abstract 861.
26. Wang M, Delasalle K, Giralt S, Alexanian R. Rapid control of previously untreated multiple myeloma with bortezomib-lenalidomide-dexamethasone (BLD). Hematology 2010;15:70-73
27. Roussel M, Avet-Loiseau H, Moreau P et al. Frontline Therapy with Bortezomib, Lenalidomide, and Dexamethasone (VRD) Induction Followed by Autologous Stem Cell Transplantation, VRD Consolidation and Lenalidomide Maintenance In Newly Diagnosed Multiple Myeloma Patients: Primary Results of the IFM 2008 Phase II Study. Blood (ASH Annual Meeting Abstracts), Nov 2010; 116: 624
28. Richardson PG, Weller E, Lonial S, et al. Lenalidomide, bortezomib and dexamethasone combination therapy in patients with newly diagnosed multiple myeloma. Blood 2010; 116:679-86
29. Kumar S et al: Novel Three- and Four-Drug Combination Regimens of Bortezomib, Dexamethasone, Cyclophosphamide, and Lenalidomide, for Previously Untreated Multiple Myeloma: Results From the Multi-Center, Randomized, Phase 2 EVOLUTION Study**.** Blood (ASH Annual Meeting Abstracts), Nov 2010; 116: 621
30. McCarthy PL, Owzar K, Hofmeister CC et al. Lenalidomide after stem-cell transplantation for multiple myeloma. N Engl J Med 2012;366:1770-1781
31. Attal M, Lauwers-Cances V, Marit G et al. Lenalidomide maintenance after stem-cell transplantation for multiple myeloma 2012;366:1782-1791.
32. Palumbo A, Hajek R, Delforge M et al. Continuous lenalidomide treatment for newly diagnosed multiple myeloma. N Engl J Med 2012;366:1759-1769.
33. Sonneveld P, Schmidt-Wolf IGH, Van der Holt B. HOVON-65/GMMG-HD4 randomized phase III trial comparing bortezomib, doxorubicin, dexamethasone (PAD) vs VAD followed by high-dose melphalan and maintenance with bortezomib or thalidomide in patients with newly diagnosed multiple myeloma. Blood 2010;116: Abstract 40.

34. Mellqvist U-H, Westin J, Gimsing P et al. Improved response rate with bortezomib consolidation after high dose melphalan: first results of a Nordic Myeloma Study Group randomized phase III trial. Blood 2009;114: Abstract 530.
35. Silvennoinen R, Kairisto V, Itälä-Remes M et al. Bortezomib plus dexamethasone (VelDex) followed by autologous stem cell transplantation (ASCT) produces molecular remission (MolR) in 23% of patients with newly diagnosed multiple myeloma (MM). Haematologica 2011;96 Suppl 1:S1-S178. Abstract#0901
36. Attal M, Harousseau JL, Stoppa AM et al. for the Intergroupe Francais du Myelome. A prospective, randomized trial of autologous bone marrow transplantation and chemotherapy in multiple myeloma. N Engl J Med 1996; 335:91–97.
37. Child JA, Morgan GJ, Davies FE, et al. High-dose therapy with hematopoietic stem-cell rescue for multiple myeloma. N Engl J Med 2003;348:1875–1883.
38. Fermand JP, Ravaud P, Chevret S, et al. High-dose therapy and autologous peripheral blood stem cell transplantation in multiple myeloma: upfront or rescue treatment? Results of a multicenter sequential randomized trial. Blood 1998;92:3131–3136.
39. Fermand JP, Katsahian S, Divine M, et al. High-dose therapy and autologous stem cell transplantation compared with conventional treatment in myeloma patients aged 55 to 65 years: long-term results of a randomized control trial from the Group Myeloma-Autogreffe. J Clin Oncol 2005;23:9227–9233.
40. Blade J, Rosinol L, Sureda A, et al. High-dose therapy intensification compared with continued standard chemotherapy in multiple myeloma patients responding to the initial chemotherapy: long-term results from a prospective randomized trial from the Spanish cooperative group PETHEMA. Blood 2005;106:3755–3759.
41. Barlogie B, Kyle RA, Anderson KC, et al. Standard chemotherapy compared with high-dose chemoradiotherapy for multiple myeloma: final results of phase III US Intergroup Trial S9321. J Clin Oncol 2006;24:929–936.
42. Attal M, Harousseau JL. Role of autologous stem-cell transplantation in multiple myeloma. Best Pract Res Clin Haematol 2007;20:747–759.
43. Attal M, Harousseau JL, Facon T, et al. Single versus double autologous stem cell transplantation for multiple myeloma. N Engl J Med 2003; 349:2495–2502.
44. Cavo M, Tosi P, Zamagni E, et al. Prospective randomized study of single compared with double autologous stem cell transplantation for Multiple Myeloma: Bologna 96 clinical study. J Clin Oncol 2007;25:2434–2441.
45. Fermand J, Alberti C, Marolleau J. Single versus tandem high dose therapy (HDT) supported with autologous blood stem cell (ABSC) transplantation using unselected or CD34-enriched ABSC: results of a two by two designed randomized trial in 230 young patients with multiple myeloma (MM). Paper presented at Xth International Myeloma Foundation Workshop; April 10–14, 2005; Sydney, Australia.
46. Goldschmidt H. Single vs. double HDT in multiple myeloma. Paper presented at Xth International Myeloma Workshop; June 28, 2007; Kos, Greece.
47. Sonneveld P, van der Holt B, Segeren CM, et al. Intermediate-dose melphalan compared with myeloablative treatment in multiple myeloma: long-term follow-up of the Dutch Cooperative Group HOVON 24 trial. Haematologica 2007;92(7):928–935.
48. Hsu AK, Quach H, Tai T, et al. The immunostimulatory effect of lenalidomide on NK-cell function is profoundly inhibited by concurrent dexamethasone therapy. Blood 2011;117:1605-1613.
49. Davies F, Baz R. Lenalidomide mode of action: linking bench and clinical findigs. Blood Rev 2010;24:Suppl1:S13-19.
50. Jantunen E, Putkonen M, Nousiainen T, et al. Low-dose or intermediate-dose cyclophosphamide plus granulocyte colony-stimulating factor for progenitor cell mobilization in patients with multiple myeloma. Bone Marrow Transplant 2003; **31**: 347-351
51. Bensinger W, DiPersio JF, McCarty JM. Improving stem cell mobilization strategies: future directions. Bone Marrow Transplant 2009; **43:**181-195.

52. Jantunen E, Varmavuo V, Juutilainen A, et al. Kinetics of blood $CD34^+$ cells after chemotherapy plus G-CSF in poor mobilizers: implications for pre-emptive plerixafor use. Ann Hematol 2012 Jan 26 e-pub.
53. Desikan KR, Barlogie B, Jagannath S, et al. Comparable engraftment kinetics following peripheral blood stem-cell infusion mobilized with granulocyte colony stimulating factor with or without cyclophosphamide in multiple myeloma. J Clin Oncol 1998; **16**: 1547-1553.
54. Kumar S, Giralt S, Stadtmauer EA, et al. Mobilization in myeloma reviseted: IMWG consensus perspectives on stem cell collection following initial therapy with thalidomide-, lenalidomide-, or bortezomib-containing regimens. Blood 2009; **114**: 1729-1735.
55. Goldschmidt H, Hegenbart U, Haas R, Hunstein W. Mobilization of peripheral blood progenitor cells with high-dose cyclophosphamide (4 g/m^2 or 7 g/m^2) and granulocyte colony stimulating factor in patients with multiple myeloma. Bone Marrow Transplant 1996; **17**: 691-697.
56. Dingli D, Nowakowski GS, Dispenzieri A, et al. Cyclophosphamide mobilization does not improve outcome in patients receiving stem cell transplantation for multiple myeloma. Clin Lymphoma Myeloma 2006; **6**: 384-388.
57. DiPersio JF, Stadtmauer EA, Nademanee A, et al. Plerixafor and G-CSF versus placebo and G-CSF to mobilize hematopoietic stem cells for autologous stem cell transplantation in patients with multiple myeloma. Blood 2009; **113**; 5720-5726.
58. Shaughnessy P, Islas-Ohlmayer M, Murphy J, et al. Cost and clinical analysis of autologous hematopoietic stem cell mobilization with G-CSF and plerixafor compared to G-CSF and cyclophosphamide. Biol Blood Marrow Transplant 2011; **17**: 729-736
59. Jantunen E, Lemoli RM. Preemptive useof plerixafor in difficult-to-mobilize patients: an emerging concept. Transfusion 2011 Oct 7 e-pub.
60. Jantunen E, Kvalheim G. Mobilization strategies in hard-to-mobilize patients with lymphoid malignancies. Eur J Haematol 2010; **85**: 463-371.
61. Horwitz ME, Chute JP, Gasparetto C et al. Preemptive dosing of plerixafor given to poor stem cell mobilizers on day 5 of G-CSF administration. Bone Marrow Transplant 2011 Nov 4 e-pub.
62. Jantunen E, Fruehauf S. Importance of graft characteristics in auto-SCT: implication for optimizing mobilization regimens. Bone Marrow Transplant 2011; **46**: 627-635.
63. Varmavuo V, Mäntymaa P, Kuittinen T, et al. Blood graft lymphocyte subsets after plerixafor injection in NHL patients mobilizing poorly with chemotherapy plus G-CSF. Transfusion 2012 Feb 5 e-pub.
64. Ludwig H, Durie BG, McCarthy P, et al. IMWG consensus on maintenance therapy in multiple myeloma. Blood 2012;119:3003-3015.
65. Neben K, Lokhorst HM, Jauch A, et al. Administration of bortezomib before and after autologous stem cell transplantation improves outcome in multiple myeloma patients with deletion 17p. Blood 2012;119:940-948.
66. Moreau P, Pylypenko h, Grosicki S, et al. Subcutaneous versus intravenous administration of bortezomib in patients with relapsed multiple myeloma: a randomized, phase 3, non-inferiority study. Lancet Oncol 2011;12:431-440.
67. Giralt S, Stadtmauer EA, Harousseau JL, et al. International myeloma working group (IMWG) consensus statement and guidelines regarding the current status of stem cell collection and high-dose therapy for mueltiple myeloma and the role of plerixafor (AMD 3100). Leukemia 2009;23:1904-1912.
68. Palumbo A, Rajkumar SV, Dimopoulos MA, et al. Prevention of thalidomide- and lenalidomide associated thrombosis in myeloma. Leukemia 2008a;22:414-423.
69. Snowden JA, Ahmedzai SH, Aschcraft J, et al. Guidelines for supportive care in multiple myeloma 2011. Br J Haemat 2011;154:76-103.
70. Rajkumar SV, Harousseau JL, Durie B et al. Consensus recommendations for the uniform reporting of clinical trials: report of the International Myeloma Workshop Consensus Panel 1. Blood 2011;117:4691-4695
