# Supplementary material for: RVD induction and autologous stem cell transplantation followed by lenalidomide maintenance in newly diagnosed multiple myeloma: a phase 2 study of the Finnish Myeloma Group
Source: Ann Hematol. 2019 Oct 31;98(12):2781–92. doi: 10.1007/s00277-019-03815-7 (PMC6900265; doi:10.1007/s00277-019-03815-7)
Supplement: Supplementary file 5 — (PDF 237 kb) [file 277_2019_3815_MOESM5_ESM.pdf]

1. Amendment 23.11.2013 Raija Silvennoinen, coordinating investigator

Kliininen lääketutkimus / Clinical trial

A prospective phase II study to assess immunophenotypic remission after three-drug induction followed by randomized stem cell mobilization, autologous stem cell transplantation and lenalidomide maintenance in patients with newly diagnosed multiple myeloma

Myelooman ensilinjan hoitotutkimus: RVD-alkuhoito, satunnaistettu kantasolujen mobilisaatio, autologinen kantasolujensiirto ja lenalidomidiylläpitohoito (FMG-MM02).
(RVD, lenalidomidi+ bortetsomibi+deksametasoni)

EudraCT number **2012-001051-39**
FIMEA **KLnro 124/2012**
PSSHP Tutkimuseettinen tmk (Ethical Board) **51//2012**
Protocol No. FMG-MM-02, Version1,0 Dated 7.6.2012

Page 1
Principal Investigators updated, Appendix 1

Page 11

-absolute lymphocyte count (ALC), platelet count and neutrophil counts at **d +15**, and at 1, 3, 6, and 12 months from the stem cell infusion

Page 12

Complete blood counts at **d + 15**, 1 mo, 3 mo, 6 mo, 12 mo from ASCT

Page 23

Lenalidomidi-ylläpitolääkityksen aloitus oli epätäsmällisesti sanottu tässä kohtaa “in 2 – 3 months…”, muualla se on sanottu ”…in 3 months after ASCT.”
Lenalidomide maintenance will be started after assessment of best response to ASCT in **3 months** after ASCT.
(Correction of similarity)

Page 28

Flowchart
16. STUDY INVESTIGATIONS
16.1 SCHEDULE OF INVESTIGATIONS
For safety reason laboratory tests regarding lever function have been added to be taken more frequently
Lisätään turvakokeita maksaan kohdistuneiden vakavien haittatapahtumien vuoksi:
“hematology” myös päivänä + 8
P –Alat, P-Afos, P-Bil, P-Krea, P-K, P-Na, B-gluk, P-CRP day + 8 and + 11 from all patients

Otettavista tutkimuksista on tehty tiivistelmä kaavion muotoon, missä näkyvät nämä lisäykset.
Laboratory tests have been added to the flowchart to every day use.
Appendix 2.

Uusi ohje /New quidance

Ohjeistus koskien mobilisaatioissa käytettävää G-CSF annosta /paino

Formulation of G-CSF doses for mobilization arms

Syklofosfamidi + filgrastiimi mobilisaatio, filgrastiimiannos 5µg/kg; biosimilaari 0.5 IU/kg		
Potilaan paino välillä<br>Weight	Filgrastiimiannos<br>dose	Biosimilaarien<br>IU annoksina
60 – 77 kg	300 µg	30 IU
78 – 107 kg	480µg	48 IU
108 – 137 kg	600µg	30 + 30 IU
138 kg –	780µg	30 + 48 IU

Pelkkä filgrastiimi mobilisaatio, filgrastiimiannos 10 µg/kg; biosimilaari 1.0 IU/kg		
Potilaan paino välillä	Filgrastiimiannos	Biosimilaarien<br>IU annoksina
60 – 68 kg	600 µg	2 x 30 IU
69 – 83 kg	780 µg	30 + 48 IU
84 – 92 kg	900 µg	3 x 30 IU
93 – 106 kg	960 µg	2 x 48 IU
107 – 121 kg	1080 µg	2 x 30 IU + 48 IU
122 – 134 kg	1260 µg	2 x 48 IU + 30 IU
135 kg –	1440 µg	3 x 48 IU

Liite 1

“A prospective phase II study to assess immunophenotypic remission after three-drug induction followed by randomized stem cell mobilization, autologous stem cell transplantation and lenalidomide maintenance in patients with newly diagnosed multiple myeloma”

Confidential

**EudraCT number**	**2012-001051-39**
**Protocol number**	**FMG-MM-02**
**Final version 1.0**	**Date: 7.6.2012**
**Sponsor**	**Kuopio University Hospital (Center of Medicine)**
**Coordinating Investigator**	**Raija Silvennoinen Kuopio University Hospital**

**Principal Investigators**	Pekka Anttila	Helsinki University Hospital
	Jouni Heiskanen	Helsinki University Hospital
	Tuomo Honkanen	Päijät-Häme Central Hospital Lahti
	Minna Lehto	Päijät-Häme Central Hospital Lahti
	Piotr Bazia	Kainuu Central Hospital
	Kristiina Kananen	Kainuu Central Hospital
	Anu Kutila	Mikkeli Central Hospital
	Mari Partio	Mikkeli Central Hospital
	Kirsi Launonen	Länsi-Pohja Central Hospital
	Mervi Putkonen	Turku University Hospital
	Anu Räsänen	Kymenlaakso Central Hospital
	Anu Sikiö	Keski-Suomi Central Hospital
	Merja Suominen	Kanta-Häme Central Hospital
	Marjaana Säily	Oulu University Hospital
	Timo Siitonen	Oulu University Hospital
	Sakari Kakko	Oulu University Hospital
	Venla Terävä	Tampere University Hospital
	Hanna Ollikainen	Satakunta Central Hospital
	Seppo Vanhatalo	Satakunta Central Hospital
	Esa Jantunen	Kuopio University Hospital
	Raija Silvennoinen	Kuopio University Hospital
	Taru Kuittinen	Kuopio University Hospital
